# The Sympathetic‐Immune Milieu in Metabolic Health and Diseases: Insights from Pancreas, Liver, Intestine, and Adipose Tissues

**DOI:** 10.1002/advs.202306128

**Published:** 2023-12-01

**Authors:** Wenran Ren, Meng Hua, Fang Cao, Wenwen Zeng

**Affiliations:** ^1^ Institute for Immunology and School of Medicine Tsinghua University and Tsinghua‐Peking Center for Life Sciences Beijing 100084 China; ^2^ Department of Neurosurgery Affiliated Hospital of Zunyi Medical University Zunyi Guizhou 563000 China; ^3^ SXMU‐Tsinghua Collaborative Innovation Center for Frontier Medicine Taiyuan 030001 China; ^4^ Beijing Key Laboratory for Immunological Research on Chronic Diseases Beijing 100084 China

**Keywords:** brain–body interaction, metabolism, neuroimmune, sympathetic

## Abstract

Sympathetic innervation plays a crucial role in maintaining energy balance and contributes to metabolic pathophysiology. Recent evidence has begun to uncover the innervation landscape of sympathetic projections and sheds light on their important functions in metabolic activities. Additionally, the immune system has long been studied for its essential roles in metabolic health and diseases. In this review, the aim is to provide an overview of the current research progress on the sympathetic regulation of key metabolic organs, including the pancreas, liver, intestine, and adipose tissues. In particular, efforts are made to highlight the critical roles of the peripheral nervous system and its potential interplay with immune components. Overall, it is hoped to underscore the importance of studying metabolic organs from a comprehensive and interconnected perspective, which will provide valuable insights into the complex mechanisms underlying metabolic regulation and may lead to novel therapeutic strategies for metabolic diseases.

## Graphical abstract

This review gives an overview of the latest research on how the sympathetic nervous system impacts key metabolic organs like the pancreas, liver, intestine, and adipose tissues. It highlights the sympathetic innervation and its interactions with the immune components, offering insights into metabolic regulation and potential therapies.

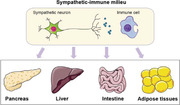

## 1 Introduction

Sympathetic innervation plays a crucial role in maintaining metabolic homeostasis by coordinating the actions of the brain and peripheral organs. Progress in neuroanatomy and functional characterization has advanced our understanding of the neural network governing metabolic activities, empowering researchers with unprecedented precision in visualizing and probing the nervous system. In parallel, the metabolic effects mediated by the sympathetic nervous system (SNS) intersect with myriads of immune cell subsets, enabling the body to synergistically detect and respond to diverse internal or environmental challenges.^[^ ^1^ ^]^ In this review, we aim to provide an overview of the evidence suggesting that: 1) sympathetic innervation is essential for regulating metabolism in individual organs, and 2) dysfunction in the immune microenvironment is tightly associated with organ pathology accompanied by sympathetic dysregulation. While this review is limited to specific organs and their particular interactions with the sympathetic and immune systems, our objective is to promote the synthesis of fundamental concepts by integrating the neuroimmune perspective. By highlighting the intricate connections between sympathetic innervation, organ metabolism, and immune function, unexpected routes might be revealed for the development of novel therapeutic strategies that target these interconnected systems.

## 2 The Sympathetic Innervation and Immune Milieu in the Pancreas

The pancreas serves as a vital organ for both endocrine and exocrine functions in maintaining physiological homeostasis. Within the pancreas, a complex network of nerve fibers encompasses various types, including spinal sensory fibers, vagal sensory nerves, autonomic fibers from the sympathetic and parasympathetic divisions, and fibers originating from the enteric nervous system (ENS) and intrapancreatic ganglia. Extensive research conducted over the years has emphasized the indispensable role of the nervous system in regulating pancreatic function.^[^ ^2^ ^]^ Emerging evidence starts to highlight the significance of its crosstalk with other systems, such as the immune system, in the modulation of pancreatic function and the pathogenesis of pancreatic disorders.

### 2.1 Sympathetic Innervation and Function in the Pancreas

**Figure 1:**
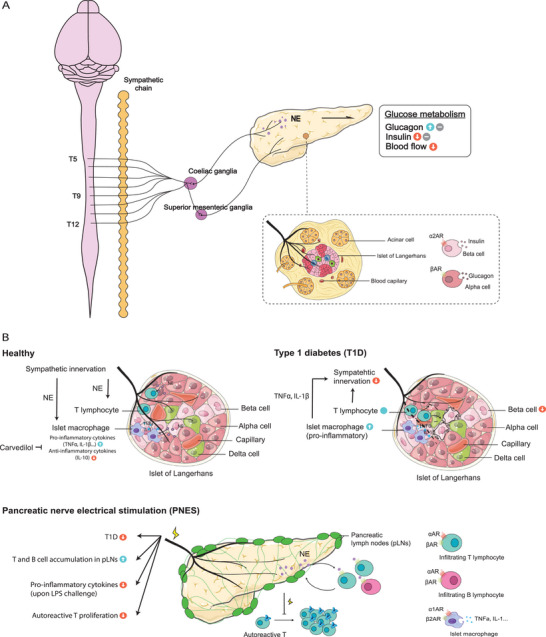
The sympathetic innervation and immune milieu in the pancreas. A) The pancreas receives sympathetic preganglionic fibers from T5‐T12 of the spinal cord, while postganglionic fibers primarily originate from the coeliac ganglia and the superior mesenteric ganglia. The sympathetic nerves project to various compartments of the organ, regulating the pancreas’ glucose metabolism. B) The sympathetic‐immune crosstalk in healthy and diseased settings of the pancreas. The pancreas harbors resident macrophages and T lymphocytes during steady state, the number of which increase in type 1 diabetes (T1D), leading to a loss of sympathetic innervation and pancreatic β‐cell damage. Pancreatic nerve electrical stimulation (PNES) has been reported to reduce the incidence of T1D, inhibit the pro‐inflammatory states following LPS stimulation, and prevent autoreactive T cell proliferation in the pancreas lymph nodes (pLNs).

The pancreas receives sympathetic innervation from preganglionic neurons located in the intermediolateral columns of the thoracic and upper lumbar segments of the spinal cord^[^ ^3^ ^]^ Postganglionic fibers innervating the pancreas primarily originate from the coeliac ganglia, with a lesser contribution from the superior mesenteric ganglia.^[^ ^4^ ^]^ Upon reaching the pancreas, these sympathetic nerves form a network of nerve fibers that innervate various compartments of the organ, including the pancreatic islets (containing insulin‐producing β‐cells, glucagon‐producing α‐cells, and other cell types), blood vessels, and exocrine acinar cells (**Figure** **1**A).^[^ ^3^, ^5^ ^]^

The distribution of sympathetic innervation within the pancreas varies across different species. In mice, sympathetic axons primarily project to the islets, where they exert a modulatory effect on hormone production.^[^ ^6^ ^]^ However, in humans, the innervation of endocrine cells within the islets is relatively sparse, and there is a preferential innervation observed surrounding the blood vessels.^[^ ^7^ ^]^

Both sympathetic and parasympathetic nerves form the neurovascular network in the pancreas and work in conjunction to maintain proper pancreatic function.^[^ ^4^, ^7^ ^]^ The sympathetic control is achieved primarily through the neurotransmitter norepinephrine (NE) and its receptors expressed on the pancreatic cells. Previous studies have shown that sympathetic stimulation generally increases blood glucose through increased glucagon and reduced insulin secretion, and decreased blood flow, while sympathetic inhibition has the opposite effects.^[^ ^3^, ^4^, ^8^ ^]^ Especially, sympathetic neurons promote islet cell migration in a β‐adrenergic‐dependent manner.^[^ ^9^ ^]^ α2A‐adrenergic receptor is the most abundant adrenergic receptor in adult mouse and human β‐cells that inhibits insulin secretion.^[^ ^10^ ^]^ β_1/2_‐adrenergic receptors are also expressed on α‐cells.^[^ ^3^, ^11^ ^]^ In the recent study using adeno‐associated virus (AAV)‐mediated targeting strategy to deliver chemogenetic constructs for cell‐type‐specific neural modulation, M. Jimenez‐Gonzalez et al. have shown that activation of pancreatic efferent parasympathetic neurons in intrinsic intrapancreatic ganglia substantially increased plasma insulin and improved glucose metabolism in male, but not in female mice, whereas the activation of pancreatic efferent sympathetic neurons in extrinsic coeliac ganglia impaired glucose metabolism. It is thus interesting for future studies to explore how the autonomic nervous system (ANS) differentially regulates the islet cells possibly in a sex‐specific manner.

In addition to autonomic efferent innervation, the pancreas receives a significant sensory afferent innervation.^[^ ^12^ ^]^ A relatively even distribution has been observed for sympathetic fibers, while sensory input shows disparate distribution of head>body>tail for both spinal and vagal afferents.^[^ ^13^ ^]^ In mice, when Alexa Fluor‐conjugated cholera toxin B was injected into the pancreas, spinal afferents were observed from T5 to T13, with the greatest contribution coming from T9‐T12. Specifically, preganglionic sympathetic inputs originate from spinal levels T5‐T12 projecting to the coeliac ganglia. The pancreatic afferents were equally distributed between right and left spinal ganglia; while the innervation from the left nodose ganglion (NG) seemed significantly greater than from the right.^[^ ^13^ ^]^ However, when AAV was injected into the pancreas, a comparable number of labeled cells were detected in the left and right NG.^[^ ^4a^ ^]^ The apparent difference might be due to the experimental method adopted in those studies and further investigation is expected to determine whether an anatomically or functionally differential pattern exists for NG when projecting to the pancreas.

**Figure 2:**
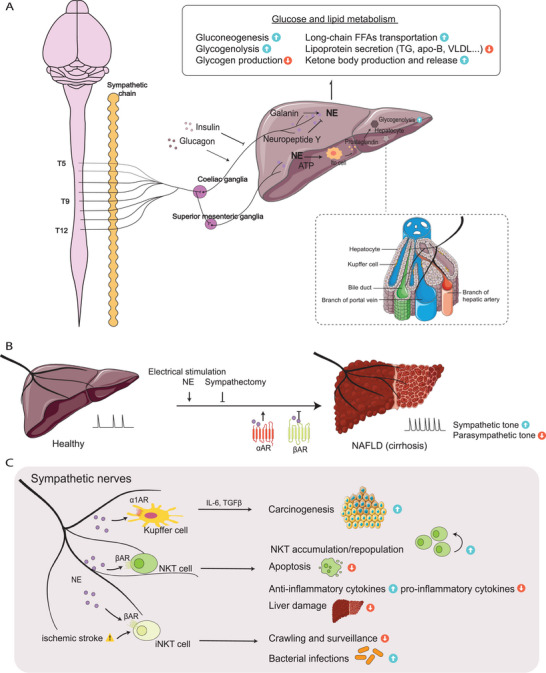
The sympathetic innervation and immune milieu in the liver. A) The liver receives sympathetic preganglionic fibers from T5‐T12, while postganglionic fibers primarily originate from the coeliac ganglia and the superior mesenteric ganglia. In rodents, the sympathetic nerves predominantly surround the haptic artery and portal veins. The sympathetic system regulates glucose and lipid metabolism in the liver mainly through norepinephrine (NE). Additional neuropeptides, such as galanin and neuropeptide Y, and circulating glucoregulatory hormones may also modulate the sympathetic effects. B) ANS imbalance, characterized by enhanced sympathetic tone and decreased parasympathetic tone, is widely reported in nonalcoholic fatty liver disease (NAFLD). Electrical stimulation of the hepatic sympathetic nerves and administration of NE exacerbate the liver damage. C) Sympathetic regulation of immune cells in the liver. The sympathetic interaction with Kupffer cells sustains an inflammatory microenvironment and is thus implicated in hepatocarcinogenesis. NE also modulates NKT cell number and cytokine production, thereby protecting the liver from immune‐mediated damage. Additionally, the sympathetic tone facilitates immunosuppressive alterations in the iNKT cell response triggered by stroke.

Overall, the coordinated activity of these neuronal networks ensures the proper function of the pancreas and maintains the delicate balance required for glucose metabolism.^[^ ^2^ ^]^

### 2.2 Sympathetic Innervation and Immune Cells in Pancreatic Function and Diseases

Pancreatic islets are intricate micro‐organs comprising not only endocrine cells but also resident antigen‐presenting cells (APCs) and T lymphocytes.^[^ ^14^ ^]^ Adrenergic receptors are expressed on a wide range of cells within both the innate and adaptive immune systems, enabling them to receive and respond to catecholaminergic signals transmitted by sympathetic nerves.^[^ ^15^ ^]^ Although only a limited number of studies have investigated the relationship between sympathetic signaling and immune modulation in the pancreas, recent research has provided evidence for the involvement of pancreatic sympathetic innervation in immune modulation and protection against autoimmune diabetes in mice (Figure 1B).^[^ ^16^ ^]^

Type 1 diabetes (T1D) is an autoimmune disease characterized by the selective destruction of pancreatic β‐cells, leading to insulin deficiency and hyperglycemia. Immune cells, particularly T lymphocytes, play a crucial role in the destruction of insulin‐producing β‐cells.^[^ ^17^ ^]^ Research in the past investigating pancreatic sympathetic innervation in both humans and animal models of T1D has yielded conflicting results. Early experiments reported the loss of sympathetic innervation in the islets of Biobreeder rats but did not observe a similar effect in streptozotocin‐induced diabetic rats.^[^ ^18^ ^]^ The study conducted by Mundinger et al. unveiled a significant and persistent reduction of sympathetic nerves, particularly in the islets of patients with both recent‐onset (<2 weeks) and long‐standing (>10 years) T1D. Importantly, this sympathetic nerve loss was not observed in individuals with type 2 diabetes (T2D).^[^ ^19^ ^]^ Consistent with these findings, Taborsky et al. provided compelling evidence of sympathetic nerve depletion within the pancreatic islets of NOD mice, an established model of autoimmune‐mediated diabetes.^[^ ^20^ ^]^ Furthermore, Campbell‐Thompson et al. reported altered adrenergic signaling and loss of sympathetic innervation in individuals with non‐diabetic islet autoantibody‐positive, but not in T1D individuals.^[^ ^3^, ^21^ ^]^ In a study utilizing the rat insulin promoter‐lymphocytic choriomeningitis virus‐glycoprotein (RIP‐LCMV‐GP) mouse model of T1D, Christoffersson et al. demonstrated a remarkable infiltration of CD8^+^ T cells in conjunction with the depletion of islet‐specific tyrosine hydroxylase‐positive (TH^+^) sympathetic nerves.^[^ ^22^ ^]^ Likewise, Taborsky et al. demonstrated a notable infiltration of islet CD3^+^ lymphocytes, which was accompanied by the loss of islet sympathetic nerves.^[^ ^23^ ^]^ Additionally, both the inhibition of sympathetic innervation and the blockade of α1‐adrenergic receptor effectively halted the autoimmune response within the pancreas, highlighting the interplay between the SNS and the immune system in the pathogenesis of T1D.^[^ ^22^ ^]^ Collectively, considering the distinct pathology of T1D and T2D and different animal models utilized in those studies, the characterization of SNS changes in diabetic conditions would benefit from further investigation by including various species, different model systems, and the full spectrum of the disease course.

The pancreas contains various types of immune subsets, both residential and from circulation. Recent studies suggest that pancreatic islets in mice harbor a distinctive subset of resident macrophages during steady state.^[^ ^24^ ^]^ Unlike the resident macrophages in the acinar exocrine tissue of the pancreas, which primarily adopt an anti‐inflammatory phenotype, these islet‐resident macrophages display a pro‐inflammatory state characterized by the expression of tumor necrosis factor‐α (TNF‐α) and interleukin‐1β (IL‐1β), even under homeostatic conditions and in the early stages of T1D.^[^ ^25^ ^]^ This pro‐inflammatory state contributes to the enhanced inflammation and damage to sympathetic nerves in the pancreas.^[^ ^25^, ^26^ ^]^ Islet macrophages, which appear to be closely associated with sympathetic fibers, are proposed to constitute the majority of APCs (up to 98%) in NOD mice.^[^ ^25^ ^]^ They also exhibit high levels of adrenergic receptors and altered cytokine production in response to adrenergic stimulation.^[^ ^25^ ^]^ Depletion of macrophages in the NOD mouse model resulted in the cessation of the autoaggressive immune response.^[^ ^27^ ^]^ Carvedilol, a β‐adrenergic receptor blocker, has been shown to significantly reduce the levels of TNF‐α, IL‐1β, interleukin‐6 (IL‐6), interleukin‐12 (IL‐12), interleukin‐17 (IL‐17), interferon‐gamma (IFN‐γ) and chemokine monocyte chemoattractant protein‐1 (MCP‐1), while increasing the anti‐inflammatory cytokine interleukin‐10 (IL‐10) in pancreatic tissue.^[^ ^28^ ^]^ Furthermore, sympathetic denervation in a dog model of acute necrotizing pancreatitis led to anti‐inflammatory signaling, as indicated by increased IL‐10 and reduced levels of TNF‐α and C‐reactive protein in the serum.^[^ ^29^ ^]^ Intriguingly, Guyot et al. found that targeting sympathetic nerves projecting into pancreatic lymph nodes (pLNs) through electrostimulation led to a reduced incidence of T1D in the NOD mouse model.^[^ ^30^ ^]^ Pancreatic nerve electrical stimulation (PNES) has been shown to induce the accumulation of B and T cells in pLNs through a β‐adrenergic receptor‐mediated mechanism.^[^ ^30^ ^]^ This stimulation also reduces pro‐inflammatory cytokine production following lipopolysaccharide stimulation.^[^ ^30^ ^]^ Additionally, PNES leads to decreased proliferation of autoreactive T cells in pLNs compared to sham controls.^[^ ^30^ ^]^ These findings suggest that targeting adrenergic receptors on immune cells can reduce pancreatic β‐cell damage and the development of T1D in mice. This therapeutic approach achieves its benefits by inhibiting inflammatory and oxidative mediators, thereby preserving β‐cell function and mitigating the onset of T1D.

While further research is necessary to fully understand the specific contributions of sympathetic innervation to immune function and the regulation of pancreatic endocrine and exocrine function, current evidence supports the close interactions between neural innervation, metabolism, and immune function in the pancreas, a re‐occurring connection observed in other metabolically important organs.

## 3 The Sympathetic Innervation and Immune Milieu in the Liver

The liver is a key organ controlling energy balance, and disruptions in liver function have major consequences for glucose and lipid homeostasis, hemodynamics, and immune processes.^[^ ^31^ ^]^ Back in the 19th century, Bernard first conducted pioneering studies on the autonomic innervation of the liver and its influence on glucose metabolism.^[^ ^32^ ^]^ During the 1960s, Shimazu et al. carried out seminal research demonstrating that electrical stimulation of the rabbit splanchnic nerves led to a significant increase in the activity of key gluconeogenic enzymes, namely glycogen phosphorylase and glucose‐6‐phosphatase.^[^ ^33^ ^]^ Those groundbreaking studies have provided important insights into the neural regulation of hepatic glucose metabolism and highlighted the interplay between the nervous system and metabolic pathways in maintaining glucose homeostasis.^[^ ^34^ ^]^

**Figure 3:**
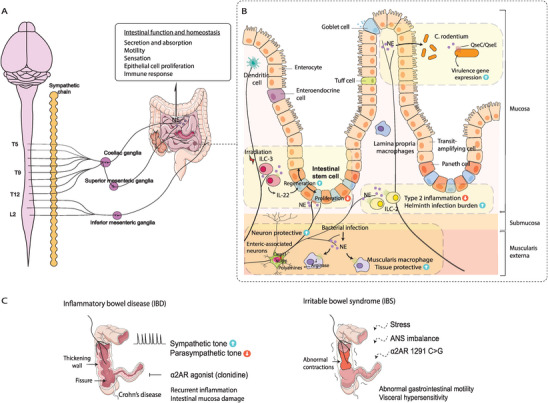
The sympathetic innervation and immune milieu in the intestine. A) The small and large intestine receives sympathetic preganglionic fibers from T5‐L2, while postganglionic fibers stem from the coeliac ganglia, the superior mesenteric ganglia, and the inferior mesenteric ganglia, modulating intestinal functions including secretion and absorption, motility, sensation, epithelial cell proliferation, and immune response. B) The sympathetic‐immune interactions in the intestine. The GI tract harbors a variety of immune cells associating closely with the sympathetic innervations. Enteric bacterial infection activates sympathetic nerves, which in turn enhance the tissue protective profile of muscularis macrophages; also through the β2‐adrenergic receptor, NE acts on ILC‐2 to mitigate type 2 inflammation. On the other hand, bacteria such as *C. rodentium* may take advantage of NE to enhance the expression of virulence genes. In addition, while NE seems to inhibit the proliferation of intestinal stem cells (ISCs) in the organoids, a recent report revealed that adrenergic nerves may enhance intestinal regeneration via ILC‐3 upon irradiation‐induced injury. C) Sympathetic dysfunction in the pathogenesis of inflammatory bowel disease (IBD) and irritable bowel syndrome (IBS). Dysregulated autonomic function, namely increased sympathetic tone and inhibited parasympathetic tone, is associated with IBD. α2‐adrenergic receptor agonist clonidine has been reported to relieve IBD symptoms. ANS imbalance has also been linked to IBS. Specifically, the α2‐adrenergic receptor 1291C>G polymorphism is significantly associated with diarrhea‐predominant IBS.

### 3.1 Sympathetic Innervation in the Liver

The sympathetic nerves that innervate the liver originate from neurons located in the coeliac and superior mesenteric ganglia. These ganglia receive inputs from pre‐ganglionic neurons situated in the intermediolateral column of the spinal cord (specifically T7‐T12) and higher brain regions in the brainstem and hypothalamus.^[^ ^35^ ^]^ Existing developmental studies on liver innervation in both human and mouse tissues indicate that sympathetic innervation initiates during late embryonic development, followed by a gradual increase in innervation density postnatally (**Figure** **2**A).^[^ ^36^ ^]^

Across a diverse range of species studied, the presence of sympathetic nerves has consistently been identified in key anatomical regions of the liver, including the hepatic artery, portal vein region, and the vicinity of the bile ducts. Notable variations also exist among the species. In rodents, the presence of sympathetic nerves has been observed predominantly in the portal tracts surrounding the hepatic artery and portal veins.^[^ ^37^ ^]^ In humans and guinea pigs, sympathetic nerve fibers directly supply nerve endings to hepatic lobules and along hepatic sinusoids.^[^ ^31e^ ^]^

Sympathetic activity in the liver has been shown to have rapid effects on circulating glucose levels by promoting glycogen breakdown to glucose (glycogenolysis), hepatic gluconeogenesis, and inhibiting glycogen production.^[^ ^38^ ^]^ The control of glycogenolysis through sympathetic regulation primarily involves the activation of α‐adrenergic receptors, the deletion of which leads to excessive glycogen accumulation.^[^ ^39^ ^]^ Additionally, neuropeptides released by sympathetic nerves may also influence hepatic glucose metabolism.^[^ ^40^ ^]^ Galanin has been shown to enhance the response to NE in the liver, resulting in elevated circulating glucose levels, while neuropeptide Y promotes glucose uptake by the liver and counteracts hepatic glucose release stimulated by glucagon and NE.^[^ ^40^ ^]^ Recent research suggests that sympathetic nerves can induce the release of prostaglandin from Ito cells, primarily mediated by NE and possibly the co‐transmitter adenosine triphosphate (ATP).^[^ ^41^ ^]^ This subsequent release of prostaglandin has been found to trigger glycogenolysis in hepatocytes.^[^ ^41^ ^]^ Furthermore, evidence suggests that circulating glucoregulatory hormones can modulate the stimulatory effects of hepatic nerves on glucose output. Insulin has been shown to inhibit the sympathetic (adrenergic) stimulation of glucose production.^[^ ^42^ ^]^ Additionally, when glucagon is present, sympathetic nerve stimulation further enhances glucose output while reducing the increased uptake of lactate mediated by glucagon.^[^ ^42^ ^]^ Moreover, denervation of the hepatic sympathetic nerves leads to a decrease in the transportation of long‐chain fatty acids into the mitochondria through carnitine palmitoyltransferase.^[^ ^31^, ^43^ ^]^ Sympathetic stimulation plays an inhibitory role in the secretion of lipoproteins, including triglycerides (TG), apolipoprotein‐B, and very low‐density lipoprotein, and promotes the production and release of ketone bodies from the liver.^[^ ^31^, ^44^ ^]^

Despite limited knowledge about the exact physiological significance of liver innervations, studies on liver‐transplanted patients have provided insights into the potential role of the ANS in liver metabolism. In liver‐transplanted patients, no reinnervation of hepatic sympathetic efferent nerves has been observed up to 30 months after transplantation, and these patients exhibit relatively normal insulin‐dependent glucose metabolism.^[^ ^37^, ^44^, ^45^ ^]^ After long‐term recovery from the initial effects of immunosuppressive treatment, protein and free fatty acid metabolism also return to nearly normal levels in liver‐transplanted patients.^[^ ^46^ ^]^ However, liver transplant recipients have been reported to experience a long‐term increase in the incidence of dyslipidemia, postprandial hyperglycemia, insulin resistance, obesity, and alterations in intrahepatic microcirculation, suggesting a potential contribution of the nervous system, particularly the autonomic nerves, to the long‐term regulation of liver metabolism.^[^ ^31^, ^47^ ^]^

Nonalcoholic fatty liver disease (NAFLD) is a progressive condition characterized by the accumulation of excessive fat in hepatocytes, often associated with obesity, diabetes, hypertension, and dyslipidemia. NAFLD encompasses a spectrum of conditions, ranging from simple hepatic steatosis to more inflammatory forms such as nonalcoholic steatohepatitis (NASH), which can lead to liver fibrosis, cirrhosis, and liver failure.^[^ ^48^ ^]^ NAFLD is highly prevalent, affecting nearly a quarter of the global population.^[^ ^49^ ^]^ Recent research has highlighted the significant role of ANS imbalance in the development and progression of NAFLD. This imbalance is characterized by increased sympathetic activity and decreased parasympathetic tone.^[^ ^50^ ^]^ Studies in mice with diet‐induced NAFLD have demonstrated a significant increase in the firing rate of liver sympathetic nerves.^[^ ^51^ ^]^ Diet‐induced hepatic steatosis is characterized by an increased sympathetic tone within the liver.^[^ ^51^, ^52^ ^]^ Patients with NAFLD have also shown increased sympathetic outflow, as evidenced by studies measuring sympathetic nerve activity.^[^ ^53^ ^]^ Autonomic dysfunction has been observed in patients with cirrhosis, with the severity of dysfunction being associated with disease progression.^[^ ^54^ ^]^ A recent investigation by Adori et al. utilized advanced 3D immunoimaging techniques to examine liver sympathetic innervation in patients with NAFLD. The study revealed that augmented sympathetic axonal sprouting during the early stage of the disease, while advanced steatohepatitis displayed substantial loss of sympathetic innervation.^[^ ^52^ ^]^ These findings indicate the important role of chronic sympathetic hyperexcitation in promoting axonal degeneration, supported by the genetic phenocopy observed in Vav3‐deficient mice.^[^ ^52^, ^55^ ^]^

Investigation on the pathological conditions of NAFLD, hepatitis, or cirrhosis has also provided evidence for how sympathetic activity could affect those disease conditions.^[^ ^56^ ^]^ Αdministration of a β‐antagonist, propranolol, worsened liver injury in a mouse model of NAFLD while chemical sympathectomy effectively reversed steatosis, independent of changes in body weight, caloric intake, or adiposity.^[^ ^52^, ^57^ ^]^ Electrical stimulation of hepatic sympathetic nerves and administration of NE led to an exacerbation of liver damage, and activation of β‐adrenergic receptors and inhibition of α‐adrenergic receptors have anti‐apoptotic effects and reduce overall liver injury in mouse models.^[^ ^58^ ^]^ Studies have demonstrated that interventions targeting the sympathetic innervation of the liver, such as pharmacological blockade or surgical denervation of the portal sympathetic bundle, can effectively reduce the excessive uptake of free fatty acids by the liver, thereby mitigating the development and progression of hepatic steatosis (Figure 2B).^[^ ^51^ ^]^

### 3.2 Sympathetic Innervation and Immune Regulation in the Liver

The liver, in addition to hepatocytes, houses a highly sophisticated immune system comprising a diverse array of cells, such as Kupffer cells (KCs), monocyte‐derived macrophages, dendritic cells (DCs), natural killer (NK) cells, natural killer T (NKT) cells, T cells, and B cells that play a critical role in maintaining liver homeostasis and participating in liver pathologies, including autoimmune hepatitis, steatohepatitis, cirrhosis, and liver cancer.^[^ ^59^ ^]^ Interestingly, these immune cells are found to be adjacent to sympathetic axons in the liver and could be regulated by sympathetic fibers through their adrenergic receptors.^[^ ^60^ ^]^ Although the precise spatial relationship between nerve structures and immune cells within the liver remains incompletely understood, extensive investigations utilizing surgical and genetic approaches have provided valuable insights into the area of neuroimmunology in liver diseases (Figure 2C).

Hepatocellular carcinoma (HCC) frequently develops in cirrhotic livers as a consequence of chronic inflammation. The SNS has been implicated in tumor initiation and metastasis. Notably, Huan et al. have shown that a high density of sympathetic nerve fibers and the presence of α1‐adrenergic receptors on KCs are associated with a poor prognosis in HCC. Their findings highlight the critical role of sympathetic innervation in hepatocarcinogenesis, wherein the SNS promotes HCC development by activating α1‐adrenergic receptors on KCs. This activation enhances KC activation and sustains the inflammatory microenvironment through the secretion of IL‐6 and transforming growth factor β (TGFβ).^[^ ^61^ ^]^ In addition, recent investigations have provided further insights into the regulatory mechanisms of bacterial phagocytosis performed by KCs in the liver, highlighting the involvement of the adrenal gland and the sympathetic outputs from the celiac ganglion.^[^ ^62^ ^]^

**Figure 4:**
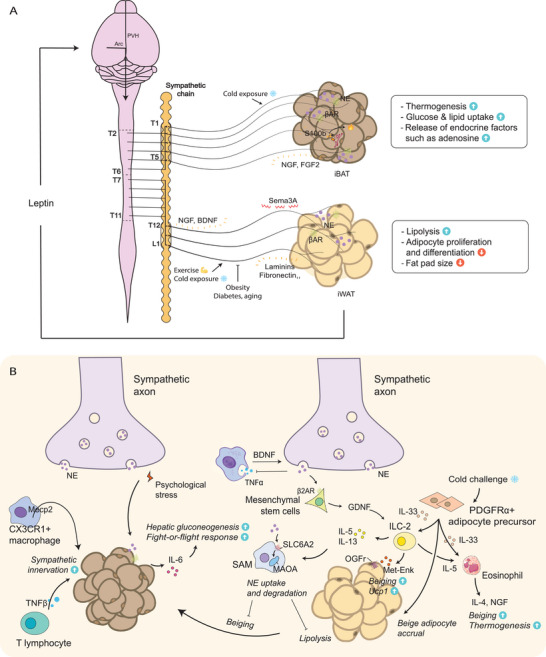
The sympathetic innervation and immune milieu in the adipose tissue. A) iWAT and iBAT receive innervation from different sections of the sympathetic chain, mediating lipolysis, adipocyte proliferation and differentiation of iWAT and thermogenesis, glucose and lipid uptake, and the release of endocrine factors in iBAT. The sympathetic axon growth is guided by various attractive and repulsive cues during innervation. Exercise and cold exposure have been reported to increase sympathetic nerve density, while obesity, diabetes, and aging may significantly reduce sympathetic innervation. Additionally, leptin signaling is reported to regulate the sympathetic architecture through a top‐down neural circuit. B) The sympathetic‐immune crosstalk in the adipose tissue. CX3CR1+ macrophages and T lymphocytes facilitate the sympathetic innervation of BAT. Macrophages also act as a source of BDNF, facilitating sympathetic axon growth; meanwhile, sympathetic nerves inhibit the TNFα level in macrophages, maintaining an anti‐inflammatory state. Sympathetic nerve‐associated macrophages (SAMs) uptake and degrade NE, thereby inhibiting beiging and lipolysis of WAT. ILC2, eosinophil, and adipose tissue macrophages interact intricately via cytokines such as IL‐5 and IL‐13 and collectively regulate beiging and thermogenesis. Additionally, in response to psychological stress, BAT produces IL‐6, thereby facilitating hepatic gluconeogenesis to support fight‐or‐flight response.

NKT cells, a distinct subset of lymphocytes arising from the thymus, play a critical role in bridging innate and adaptive immune responses. These cells possess a limited T cell receptor repertoire, primarily composed of Vα24 and Vβ11 chains in humans, and recognize antigens in association with the MHC class I‐like molecule CD1d. Within the liver, NKT cells constitute a significant proportion of lymphocytes, accounting for up to 30% of the intrahepatic lymphocyte population.^[^ ^63^ ^]^ However, in ob/ob mice, there is a postnatal depletion of hepatic NKT cells due to increased apoptosis.^[^ ^64^ ^]^ Notably, treatment with NE inhibits apoptosis and restores hepatic NKT cell numbers. Surgical procedures involving sympathetic nerve excitation have also been identified as factors that promote NKT cell repopulation. Studies have demonstrated that NKT cell accumulation in the liver during regeneration is associated with sympathetic nerve excitation, which can be attenuated by the administration of propranolol.^[^ ^65^ ^]^ In addition to their effects on NKT cell numbers, sympathetic nerves can modulate the functional behavior of these cells within the liver. Treatment with NE has been shown to reduce the production of pro‐inflammatory cytokines by NKT cells, while concurrently increasing the production of anti‐inflammatory cytokines.^[^ ^64^ ^]^ Consequently, this shift in cytokine production mitigates hepatotoxicity, protecting liver tissue from immune‐mediated damage. In mice, ischemic stroke elicits notable immunosuppressive alterations in hepatic invariant natural killer T cells (iNKT cells) via noradrenaline signaling.^[^ ^66^ ^]^ iNKT cell response triggered by stroke is substantially reversed by chemical depletion of sympathetic neurons through 6‐OHDA or administration of propranolol.^[^ ^66^ ^]^ Notably, mice subjected to 6‐OHDA or propranolol treatment exhibited a considerable reduction in spontaneous bacterial infections at the 24‐h post‐stroke timeframe.^[^ ^66^ ^]^ These findings underscore the critical role of sympathetic innervation in influencing the functional phenotype of NKT cells in the liver.^[^ ^64^ ^]^

In addition to KC and NKT, other immune cells are also suggested to interact with SNS and regulate liver diseases. For instance, recent studies have suggested that NE released from hepatic sympathetic nerves plays a crucial role in protecting the liver from Fas‐mediated fulminant hepatitis and promoting liver regeneration through the activation of the sympathetic nerve‐group 1 innate lymphoid cells (ILC1)‐interleukin‐22 (IL‐22) axis.^[^ ^67^ ^]^

Collectively, these findings suggest a bidirectional relationship between sympathetic nerves and immune cells in the liver. However, the precise outcome and underlying mechanisms of differential signaling through adrenergic receptors in the immune response to metabolic stress remain unknown, representing an area for future investigation. Further studies are needed to better understand the precise neuroimmune interactions and their contributions to the pathogenesis of metabolic liver disorders.^[^ ^14^, ^68^ ^]^

## 4 The Sympathetic Innervation and Immune Milieu in the Intestine

The intestine is covered by a vast mucosal surface and harbors the largest immune reservoir within the body. It comprises various cell types, including absorptive epithelial cells, neuroendocrine cells, Paneth cells, goblet cells, immune cells, and more. Each of these cell types contributes to essential functions such as absorption, neuroendocrine signaling, digestion, and immune responses.^[^ ^69^ ^]^ The gastrointestinal (GI) tract is innervated by both intrinsic ENS and extrinsic fibers, which include visceral sensory afferent and visceral efferent (sympathetic and parasympathetic) fibers. The intricate network of nerves collectively regulates the GI tract. The maintenance of intestinal homeostasis relies on the coordinated interaction between the nervous and immune systems, enabling the sensing and appropriate response to luminal contents. Numerous studies have documented that the ANS, both directly and indirectly, regulates the physiological activity of various intestinal cell types, including intestinal epithelial cells (IECs), stromal cells, enteric nerves, and intestinal immune cells. Adrenergic receptors are widely expressed in different intestinal cell types, indicating their involvement in the regulation of intestinal functions. In this section, our focus will be on the innervation of the SNS and its interplay with intestinal immune cells under both physiological and pathological conditions.

### 4.1 The Sympathetic Innervation in the Intestine

The presence of sympathetic nerves in the GI tract was initially observed as early as the 1940s,^[^ ^70^ ^]^ Using fluorescent microscopy, Jacobowitz investigated adrenergic fibers in the small intestine of cats and monkeys, revealing the presence of nerve fibers extended up to the tunica propria.^[^ ^71^ ^]^ These fibers exhibited branching patterns over the basal aspect of the glandular epithelium.^[^ ^71^ ^]^ Additionally, Gabella and Costa utilized the Falck‐Hillarp method, which enables the histochemical detection of biogenic amines, to detect the distribution of adrenergic nerve fibers in the mucosa of various segments of the digestive tract in guinea pigs. Adrenergic innervation was seen in the mucosa, particularly around the crypt epithelium.^[^ ^72^ ^]^

Sympathetic neurons that innervate the GI tract have their cell bodies located in the sympathetic chain (paravertebral) ganglia, as well as the large prevertebral ganglia located in the abdomen and pelvis.^[^ ^73^ ^]^ Specifically, the coeliac‐mesenteric ganglia extend fibers to the stomach, small intestine, and to some extent, the proximal large intestine. The fibers projecting to the large intestine originate from the inferior mesenteric ganglia, while those innervating the rectum originate from the pelvic ganglia. The innervation from these prevertebral ganglia forms an extensive network of fibers that supply the smooth muscle wall, ganglia of the myenteric plexus (MP) and submucosal plexus (SMP), and arteries within the GI tract. Sympathetic nerve endings rich in NE provide extensive innervation to various regions of the intestine, including the serosa, mucosa, muscularis, myenteric plexus, and Peyer's patches.^[^ ^74^ ^]^ This network plays a crucial role in regulating intestinal function and maintaining homeostasis (**Figure** **3**A).^[^ ^75^ ^]^

The early research of developmental biology has revealed the growth pattern of axons from sympathetic neurons, indicating their directional growth along arteries.^[^ ^76^ ^]^ In murine models at embryonic stages E13.5−E15.5, it has been observed that sympathetic axons extend along arteries in the mesentery and subsequently penetrate the gut wall after E15.^[^ ^77^ ^]^ Immunodetection of TH reveals that these axons originate from the paravertebral ganglia and prevertebral ganglia, traverse the gut mesentery, and innervate the entire gastrointestinal tract from E12.5 to E16.5.^[^ ^78^ ^]^ Interestingly, mesenteric arteries not only serve as a conduit for the innervation of the gut but also become targets for innervation themselves. Notably, under the guidance of netrin‐1, the development of sympathetic innervation in mesenteric arteries occurs postnatally, following the projection of sympathetic axons into the gut.^[^ ^79^ ^]^

Through interaction with the α‐adrenergic receptor, sympathetic nerves‐derived NE modulates a range of digestive functions, such as secretions and motility,^[^ ^80^ ^]^ gastro‐colonic sensation,^[^ ^81^ ^]^ and epithelial cell proliferation.^[^ ^82^ ^]^ Notably, the concentration of the sympathetic nerve endings from the SNS is particularly high at the base of the crypt, which coincides with the location of intestinal stem cells (ISCs).^[^ ^72^, ^83^ ^]^ ISCs have been found to express α2A‐adrenergic receptors, and studies have shown that the application of NE to small intestine organoids leads to a decrease in proliferation.^[^ ^84^ ^]^ Additionally, Caco‐2 cells engineered to express α2A‐adrenergic receptors showed increased peptide absorption in response to NE.^[^ ^84b^ ^]^ Hansen et al. conducted a study that demonstrated the inhibitory effect of electrical nerve stimulation on GLP‐1 secretion, leading to reduced glucose sensitivity in animals. Interestingly, this inhibitory effect was abolished when phentolamine (an α‐adrenergic receptor antagonist) was administered, indicating that sympathetic α‐adrenergic signals establish an inhibitory tone on the release of GLP‐1 in the ileum.^[^ ^85^ ^]^ In 2017, Haber et al. conducted a comprehensive single‐cell RNA sequencing study of the small intestinal epithelium, which revealed the widespread presence of α2A‐adrenergic receptors in various types of gut epithelial cells, including ISCs and enterocytes, consistent with previous studies.^[^ ^86^ ^]^

#### 4.1.1 SNS Dysfunction in Inflammatory Bowel Disease

The SNS not only maintains homeostasis but also plays a significant role in the pathogenesis of the intestine. Inflammatory molecules can activate the sympathetic reflex, leading to compromised gastrointestinal motility and influencing the intestinal immune system.^[^ ^87^ ^]^ This dynamic involvement of the SNS has been observed in various conditions, such as intestinal parasitic infections and inflammatory bowel disease (IBD).^[^ ^1^, ^88^ ^]^

IBD encompasses a group of chronic inflammatory disorders of the GI tract, primarily including Crohn's disease (CD) and ulcerative colitis (UC).^[^ ^89^ ^]^ These conditions are characterized by recurrent inflammation and damage to the intestinal mucosa, leading to a range of symptoms and possible complications. Altered neuronal regulation is associated with IBD, resulting in dysregulated autonomic function.^[^ ^90^ ^]^ During active IBD, there is evidence of decreased parasympathetic influence and increased sympathetic influence.^[^ ^91^ ^]^ Rodent models of colitis induced by 2,4,6‐trinitrobenzene sulfonic acid, dextran sodium sulfate, or T. spiralis. have shown altered gut sympathetic function.^[^ ^92^ ^]^ In patients with CD and UC, autonomic hyperreflexia is significantly associated with more severe inflammation and systemic manifestations of the disease.^[^ ^93^ ^]^ Interestingly, the administration of an α2‐adrenergic receptor agonist has been found to restore normal autonomic cardiovascular function and improve the IBD disease activity index scores,^[^ ^91b^ ^]^ consistent with previous studies on the beneficial effects of clonidine treatment for IBD.^[^ ^90^, ^94^ ^]^ The mechanisms underlying this reduction in the evoked release may involve increased levels of the autoinhibitory presynaptic α2‐adrenergic receptor^[^ ^95^ ^]^ or decreased voltage‐gated calcium currents.^[^ ^90^, ^92^ ^]^ These observations highlight the intricate interplay between autonomic dysfunction, disease severity, and the potential for targeted pharmacological interventions to alleviate IBD symptoms (Figure 3C).

#### 4.1.2 SNS Dysfunction in Irritable Bowel Syndrome

Irritable bowel syndrome (IBS) is a common functional gastrointestinal disorder characterized by recurrent abdominal pain accompanied by changes in bowel habits. In addition to pain, many patients with IBS experience non‐painful abdominal discomfort and may also have comorbid psychiatric conditions such as anxiety and depression.^[^ ^96^ ^]^ This chronic condition significantly impairs the quality of life for those affected. The precise cause of IBS remains unclear, and it is believed to result from a complex interplay of genetic, environmental, and psychosocial factors. IBS is a significant global health concern, with prevalence rates ranging from 1.1% to 45% worldwide, and between 5% and 10% in most Western countries and China.^[^ ^96^, ^97^ ^]^

The increased activation of the SNS plays a significant role in the response to stress, and chronic life stressors have been found to precede the onset and/or worsening of symptoms in individuals with IBS.^[^ ^98^ ^]^ As early as 1928, a hypothesis was proposed linking IBS dysfunction to the ANS.^[^ ^99^ ^]^ Several studies have provided evidence of ANS dysfunction or imbalance in individuals with IBS, which contributes to abnormal gastrointestinal motility and visceral hypersensitivity.^[^ ^100^ ^]^ An increasing body of evidence suggests the presence of sympathetic adrenergic dysfunction in a specific subgroup of individuals with IBS,^[^ ^101^ ^]^ which impacts both the motor and sensory functions of the GI tract in humans.^[^ ^102^ ^]^ Notably, α2‐adrenergic receptors have shown prominent effects in the human colon. These receptors, particularly the prejunctional α2A‐adrenergic receptor, play a central role in regulating systemic sympathetic activity through negative feedback at presynaptic nerve endings.^[^ ^103^ ^]^ Polymorphisms in the α2A‐adrenergic receptor gene have been associated with constipation and high somatic symptoms in patients with bowel diseases characterized by low bowel functioning.^[^ ^104^ ^]^ and the α2A‐1291C > G polymorphism has been significantly associated with diarrhea‐predominant IBS (D‐IBS).^[^ ^105^ ^]^ However, our understanding of the involvement and underlying mechanisms of sympathetic nerves in the development and progression of IBS is still in its early stages and requires further investigation (Figure 3C).^[^ ^106^ ^]^

### 4.2 Sympathetic Innervation and Immune Cells in Intestine

The GI tract contains a wide range of neuronal and immune cell types, allowing for precise and specialized responses to specific stimuli.^[^ ^107^ ^]^ Within this cellular diversity, sympathetic signals have been shown to play a role in regulating immune responses in the intestine through the release of NE. This regulatory function extends to various immune cell populations, including macrophages, lymphocytes, mast cells, DCs, and innate lymphoid cells (ILCs), as adrenergic receptors are expressed by most innate and adaptive immune cells (Figure 3B).^[^ ^108^ ^]^

Macrophages are highly prevalent within the resting lamina propria and between the circular and longitudinal muscle layers of the muscularis externa in the GI tract. The study by Gabanyi et al. utilizing imaging and transcriptional profiling techniques probed the involvement of sympathetic innervation in the GI tract and identified potential mechanisms through which the ENS spatially and immunologically influences intestinal macrophages.^[^ ^109^ ^]^ Distinct populations of macrophages within the gut exhibit specific phenotypes and functions. Lamina propria macrophages share similarities with pro‐inflammatory macrophages, while muscularis macrophages display a tissue‐protective program resembling alternatively activated macrophages. In vitro studies have shown that peritoneal macrophages, when cultured with enteric neurospheres, adopt a phenotype similar to that of muscularis macrophages, an effect mediated by the engagement of the β2‐adrenergic receptor.^[^ ^109^ ^]^ Importantly, muscularis macrophages also play a role in preventing neuronal death post‐infection, which is mediated by a Nlrp6‐ and Casp11‐dependent mechanism.^[^ ^110^ ^]^ Activation of extrinsic sympathetic ganglia through enteric bacterial infection enhances the tissue‐protective profile of muscularis macrophages in protecting intrinsic enteric‐associated neurons, notably through an arginase‐1‐polyamine axis via NE signaling.^[^ ^74^, ^110^, ^111^ ^]^ Additionally, ex vivo catecholaminergic treatment of Peyer's patches has shown decreased rates of Salmonella translocation, suggesting a potential role of adrenergic signaling in mitigating infection.^[^ ^112^ ^]^ These findings suggest that functional crosstalk between neurons and macrophages in the intestine may contribute to the long‐term symptoms observed in infection, IBS, or IBD.

Type 2 immune responses have evolved as a defense mechanism against helminthic infections, and their dysregulation can contribute to the development of chronic intestinal inflammatory disorders.^[^ ^16a^ ^]^ Among the key regulators of type 2 immune responses are T helper 2 cells (Th2) and group 2 innate lymphoid cells (ILC2s), which play pivotal roles in orchestrating various inflammatory processes, including tissue repair, metabolic homeostasis, allergic inflammation, and anti‐helminth parasite immunity.^[^ ^113^ ^]^ Recent research has shed light on the existence of neuron‐ILC2 units, highlighting the involvement of a neuron‐based regulatory circuit that influences ILC2 activity in multiple physiological processes within the intestine.^[^ ^114^ ^]^ ILC2s express a variety of receptors for neuropeptides including the β2‐adrenergic receptor, indicating their responsiveness to neuronal signals within the gut microenvironment. Intriguingly, ILC2s have been found to colocalize with adrenergic neurons in the intestine.^[^ ^113a^ ^]^ Inhibition of the β2‐adrenergic receptor pathway has been shown to enhance ILC2 responses, promote type 2 inflammation, and reduce the burden of helminth infections. Conversely, treatment with β2‐adrenergic receptor agonists has been found to exacerbate the infection burden in mouse models of helminthic infection.^[^ ^113a^ ^]^ And β‐adrenergic receptor signaling drives IL‐22 production from type 3 innate lymphoid cells (ILC3s) post irradiation induced injury to promote epithelial regeneration.^[^ ^115^ ^]^ These collective findings strongly suggest that intestinal ILCs integrate neuronal signals, particularly those mediated by adrenergic neurons, to critically shape their responses and maintain tissue homeostasis and defense.

Interestingly, it has been discovered that bacteria possess functional adrenergic receptors, namely QseC and QseE, which enable them to respond to epinephrine and NE and induce the expression of virulence genes, the locus of enterocyte effacement (LEE), in enteric pathogens.^[^ ^116^ ^]^ Notably, pathogenicity is significantly reduced in mutant strains of *Citrobacter rodentium* that are deficient in QseC and QseE. It is worth mentioning that *C. rodentium* exhibits a decreased ability to colonize dopamine β‐hydroxylase‐deficient mice, which are unable to produce epinephrine and NE.^[^ ^117^ ^]^ This intriguing finding implicates a link between the catecholamine biosynthetic pathways in the host and bacterial infections, offering potential novel strategies for combating intestinal bacterial infections that are independent of antibiotics.

The comprehensive understanding of the sympathetic‐adrenergic receptor axis in the GI tract and its impact on gut homeostasis introduces a new dimension of intricacy to our knowledge of neuroimmune interactions. In‐depth studies have offered exciting prospects for the advancement of novel therapeutic strategies targeting the adrenergic pathways in various intestinal disorders.

## 5 The Sympathetic Innervation and Immune Milieu in Adipose Tissues

Over the past few decades, the perception of adipose tissue has shifted from that of a passive energy storage depot to a dynamic and metabolically active endocrine organ.^[^ ^118^ ^]^ Adipose tissue is now recognized as playing crucial roles in systemic glucose and energy homeostasis, insulin resistance, the immune response, and thermogenesis.^[^ ^119^ ^]^ This recognition has been further complicated by the realization that adipose tissue is a highly heterogeneous community, comprising various cell types such as adipocytes, adipocyte precursor cells, innervating neurons, and immune cells.^[^ ^120^ ^]^ The advancement in high‐resolution techniques like single‐cell RNA sequencing (scRNA‐seq) and advanced 3D imaging has provided new insights into the complexity of adipose tissue. These techniques have enabled a deeper understanding of the cellular subtypes and their respective roles within adipose tissue, while also raising new questions for further exploration.^[^ ^121^ ^]^

Adipocytes, the primary cell type in adipose tissue, exist in distinct depots known as white adipose tissue (WAT) and brown adipose tissue (BAT).^[^ ^122^ ^]^ WAT, also referred to as white fat, is the predominant form of adipose tissue in adults. It serves as a storage site for excess energy in the form of TG. During times of energy surplus, WAT undergoes lipogenesis to accumulate TG. Conversely, during energy deficits, WAT releases stored TG through lipolysis to provide fuel for other tissues. Additionally, WAT secretes adipokines, bioactive molecules that regulate metabolism, inflammation, and insulin sensitivity. BAT, also known as brown fat, is a specialized thermogenic tissue that generates heat. It is more abundant in newborns and hibernating mammals. BAT is characterized by multilocular adipocytes that contain a high number of mitochondria and express a unique protein called uncoupling protein 1 (UCP1). UCP1 plays a vital role in uncoupling oxidative phosphorylation, leading to the dissipation of energy as heat instead of ATP production. Cold exposure and SNS activation stimulate BAT activation. Recent studies have also suggested that BAT contributes to whole‐body energy expenditure, glucose homeostasis, and lipid metabolism. In this section, our focus centers on the intricate distribution of sympathetic nerves within adipose tissue and their dynamic interplay with immune cells during metabolic regulation.

### 5.1 The Sympathetic Innervation in the White Adipose Tissues

Adipose tissue is innervated by sympathetic fibers, which play a crucial role in mediating lipolysis, thermogenesis, and glycemic control within adipose tissue.^[^ ^123^ ^]^ The development of sympathetic innervation in inguinal WAT (iWAT) initiates during embryogenesis, and its establishment occurs between postnatal day 6 and day 28 in mice.^[^ ^124^ ^]^ Animal studies have revealed that sympathetic axons extend from the paravertebral sympathetic ganglia located along the spinal cord to reach iWAT depots. Huesing et al. employed pseudorabies virus (PRV) mutant expressing an engineered green fluorescent protein (PRV‐GFP) injections into iWAT of sympathetic TH‐tomato reporter mice and employed whole cleared torsos containing the spinal cord and paravertebral ganglia for imaging.^[^ ^125^ ^]^ The findings indicated postganglionic innervation originating from sympathetic ganglia T12‐L1 and preganglionic inputs from the intermediolateral cell column (IML) of T7‐T11 with some cases exhibiting labeled preganglionic neurons from T5‐T12 and postganglionic neurons from T7‐L2 (**Figure** **4**A).^[^ ^125^ ^]^

During the process of innervation, sympathetic axons navigate through the extracellular matrix, blood vessels, and adipocyte clusters to establish connections within WAT. This axon growth is guided by attractive and repulsive cues, including chemotropic factors and extracellular matrix molecules. Semaphorin 3A (Sema3A) is one such guidance molecule that binds to neuropilin‐1 (NP1) and activates transmembrane Plexin, triggering a repulsive signal in axon guidance.^[^ ^126^ ^]^ Sema3A is produced by smooth muscle cells of arteries and white adipocytes, while NP1 is present on perivascular and parenchymal nerves.^[^ ^127^ ^]^ Various ECM molecules in WAT also contribute to the growth and guidance of sympathetic axons, including laminins, fibronectin, collagen, and hyaluronan, which provide structural support and guidance cues for axon pathfinding.^[^ ^128^ ^]^ Neurotrophic factors play a vital role in promoting the growth and survival of sympathetic neurons. These factors, such as nerve growth factor (NGF) and brain‐derived neurotrophic factor (BDNF) can influence the development and plasticity of WAT nerves, supporting their growth and establishing connections.^[^ ^14^, ^121^, ^129^ ^]^

Traditional immunostaining methods used in thin tissue slices provided limited information about the degree of sympathetic innervation.^[^ ^130^ ^]^ However, the development of light‐sheet imaging and tissue‐clearing techniques has enabled the visualization of entire sympathetic arborizations and the identification of neuro‐adipose junctions within WAT. By employing tissue‐clearing methods, researchers have successfully detected nerve bundles throughout the iWAT, visualizing the complete axonal tree of sympathetic neurons in situ.^[^ ^14^, ^123^, ^129^ ^]^ Catecholaminergic reporter mice labeled with lipophilic dye and analyzed using two‐photon microscopy revealed that sympathetic nerves innervate fat pads and bouton‐like structures were found to innervate around 8% of adipocytes.^[^ ^123e^ ^]^

Decades of research have provided evidence supporting the role of sympathetic activity in regulating lipolysis and fat mobilization.^[^ ^130^ ^]^ Ablation of sympathetic nerves has been shown to inhibit lipolysis, leading to increased fat pad size, adipocyte proliferation, and differentiation.^[^ ^123^, ^131^ ^]^ These effects are primarily mediated by NE acting on β‐adrenergic receptors in adipose tissue. Mice lacking all three β‐adrenergic receptors exhibit significant obesity when exposed to a high‐fat diet.^[^ ^132^ ^]^ Furthermore, studies employing optogenetic stimulation of sympathetic neurons in iWAT have demonstrated an acute increase in lipolysis, resulting in a reduction in fat pad size over a 4‐week stimulation period.^[^ ^123e^ ^]^ Thus, these findings provide evidence for the functional role of sympathetic innervation in WAT and its involvement in the regulation of lipolysis and fat metabolism.

### 5.2 The Sympathetic Innervation in the Brown Adipose Tissues

Similar to WAT, sympathetic fibers are present in the vasculature and parenchyma of BAT, at a much higher density.^[^ ^123^, ^133^ ^]^ Studies utilizing PRV retrograde tracing have provided insights into the innervation pattern of interscapular BAT (iBAT). Sympathetic preganglionic innervation to iBAT originates from T2‐6 sympathetic ganglia, while post‐ganglionic projections arise from the caudal stellate ganglion (T1) and T2‐5 sympathetic chain ganglia.^[^ ^134^ ^]^ The high innervation of BAT is thought to be influenced by the secretion of tissue‐specific growth factors and the activity of key regulators. NGF and fibroblast growth factor 2 (FGF2) have been implicated in promoting sympathetic innervation and the expansion of preadipocytes in BAT.^[^ ^135^ ^]^ Calsyntenin 3b, an endoplasmic reticulum (ER)‐membrane‐bound protein selectively expressed in brown adipocytes, plays a role in promoting sympathetic innervation and thermogenesis in BAT through the secretion of the growth factor S100b.^[^ ^133b^ ^]^ PRDM16, a pivotal transcriptional co‐regulator of brown and beige fat cell determination and differentiation.^[^ ^133^, ^136^ ^]^ PRDM16 not only governs the development of brown adipocytes but also orchestrates the recruitment and maturation of sympathetic neurons within BAT, leading to enhanced sympathetic innervation and subsequent activation (Figure 4A).^[^ ^137^ ^]^

The significance of sympathetic stimulation in maintaining body temperature and increasing thermogenesis has been recognized since its initial descriptions.^[^ ^138^ ^]^ Studies utilizing rodent models have demonstrated that denervation of BAT leads to the loss of UCP1 expression, a key regulator of thermogenesis, along with decreased metabolic markers such as lipoprotein lipase activity, thermogenic mitochondrial protein activity, and glucose membrane transporter density.^[^ ^139^ ^]^ The activation of β‐adrenergic receptors, particularly β3‐adrenergic receptor, drives BAT thermogenesis and enhances glucose uptake in both rodent models and humans.^[^ ^140^ ^]^ β3‐adrenergic receptor, upon binding to NE, activates adenylate cyclase, resulting in the production of cyclic adenosine monophosphate (cAMP).^[^ ^141^ ^]^ Subsequent stimulation of protein kinase A (PKA) activity leads to the phosphorylation and activation of hormone‐sensitive lipase (HSL) and the transcription factor CREB.^[^ ^141^ ^]^ The resulting upregulation of mitochondrial biogenesis mediated by PGC‐1α and the uncoupling of the electron transport chain facilitated by UCP1 contribute to elevated heat generation and enhanced energy expenditure. This process utilizes long‐chain fatty acids produced through lipolysis to facilitate proton conductance.^[^ ^119^, ^142^ ^]^

Recent advancements in optogenetic techniques have further highlighted the role of sympathetic innervation in BAT thermogenesis. Optogenetic stimulation of sympathetic neurons innervating mouse BAT induced thermogenesis, increasing core body temperature and Ucp1 mRNA expression, without any accompanying changes in body weight.^[^ ^14^, ^143^ ^]^ Collectively, these findings emphasize the crucial role of sympathetic innervation in regulating BAT thermogenesis.

Emerging evidence suggests that the sympathetic innervation of BAT plays a crucial role not only in promoting thermogenesis but also in influencing whole‐body metabolism. Sympathetic nerves in BAT facilitate the import of excess glucose and lipids from the bloodstream, contributing to the regulation of systemic energy balance.^[^ ^144^ ^]^ Furthermore, BAT has been found to produce factors with endocrine functions that act on peripheral tissues.^[^ ^145^ ^]^ For example, a study showed that nucleotide adenosine can be released by brown adipocytes stimulated by the sympathetic nerves and adenosine‐A2A receptor signaling in BAT can protect mice from diet‐induced obesity.^[^ ^146^ ^]^ However, despite the growing understanding of the involvement of BAT in these non‐thermogenic functions, the specific contribution of the SNS to these processes remains poorly understood. Investigating how homeostatic information is encoded and transmitted by sympathetic signals could provide valuable insights into the mechanisms underlying both the thermogenic and non‐thermogenic functions of BAT.^[^ ^14^ ^]^

Sympathetic innervation is subjected to the signals within the adipose tissues and from the central nervous system. Exposure to cold temperatures or engaging in physical exercise has been shown to result in an elevation in nerve density within adipose tissue.^[^ ^129^, ^147^ ^]^ In contrast, in conditions associated with metabolic dysregulation, such as obesity, diabetes, and aging, adipose tissue sympathetic innervation is significantly reduced.^[^ ^123^, ^148^ ^]^ In addition, Wang et al. have reported that leptin signaling regulates the plasticity of sympathetic architecture in adipose tissue through a top‐down neural pathway crucial for energy homeostasis.^[^ ^148^ ^]^ They observed impaired enhancement of sympathetic innervation in both inguinal subcutaneous WAT and BAT following central disruption of BDNF neuron signaling. Interestingly, the presence of BDNF in the brain, but not in adipose tissue, suggests the possibility of additional centrally mediated regulation of sympathetic innervation in adipose tissue.^[^ ^149^ ^]^ Understanding the dynamic interplay between sympathetic activity, adipose tissue remodeling, and metabolic health is essential for unraveling the mechanisms underlying metabolic disorders.

### 5.3 Sympathetic Innervation and Immune Cells in the Adipose Tissues

The adipose function is intricately influenced by neuronal signals and immune responses.^[^ ^74^, ^121^, ^123^ ^]^ In addition, the immune cell function in adipose tissue is tightly linked to sympathetic innervation by expressing adrenergic receptors, specifically α‐ and β‐adrenergic receptors, which allow them to respond to sympathetic adrenergic signaling.^[^ ^150^ ^]^ The interaction between sympathetic innervation and immune cells in adipose tissue plays a critical role in the regulation of adipose tissue homeostasis and the development of metabolic disorders (Figure 4B).

Adipose tissue macrophages represent a substantial portion of the immune cell population within adipose tissue.^[^ ^151^ ^]^ CX3CR1^+^ macrophages appear to regulate sympathetic innervation of BAT through the transcription factor methyl‐CpG binding protein 2 (Mecp2).^[^ ^151b^ ^]^ Mice with Mecp2‐deleted CX3CR1^+^ macrophages develop obese phenotypes, thermogenic dysfunction of BAT, and resulted decrease in whole‐body energy expenditure at a steady state due to reduced NE input by impaired local sympathetic innervation.^[^ ^151^, ^152^ ^]^ Mechanistically, Plexin A4 overexpressed in Mecp2‐deleted CX3CR1^+^ macrophages seemed to interact with and block the outgrowth of Semaphorin 6A‐positive sympathetic axons in the tissue.^[^ ^151^, ^153^ ^]^

Disruption of this circuit in BAT has been associated with metabolic imbalance, demonstrating that tissue‐resident macrophages act as intermediaries between neuronal cues and the maintenance of tissue homeostasis.^[^ ^151b^ ^]^ The study by Blaszkiewicz et al. found the role of myeloid cells residing in iWAT as a source of BDNF, which serves as a local peripheral nerve survival factor. Silencing these macrophages led to a state of “genetic denervation” in iWAT, resulting in increased adiposity, reduced energy expenditure, and impaired response of UCP1 to cold stimulation.^[^ ^14^, ^147^ ^]^ In addition, sympathetic nerves could maintain an anti‐inflammatory state in mice by inhibiting TNF‐α levels in macrophages.^[^ ^154^ ^]^ The study by Pirzgalska et al.,^[^ ^155^ ^]^ found a distinct population of macrophages closely associated with sympathetic axons (sympathetic nerve‐associated macrophages) in white adipose tissue exhibit unique characteristics including the functional expression of metabolic machinery required for the uptake (SLC6A2) and degradation (MAOA) of NE, inhibiting the beiging and lipolysis of WAT.^[^ ^155^, ^156^ ^]^ The age‐related reduction in lipolysis is associated with higher gene expression levels of catecholamine‐degrading enzymes, providing a possible explanation for reduced lipolysis in advanced age.^[^ ^157^ ^]^

While macrophages dominate the immune landscape of adipose tissue, other immune cell subsets, including DCs,^[^ ^158^ ^]^ neutrophils,^[^ ^159^ ^]^ mast cells,^[^ ^160^ ^]^ and NK cells,^[^ ^161^ ^]^ also accumulate in adipose tissue during weight gain. Additionally, subsets such as ILCs are resident in adipose tissues and play important roles against obesity.^[^ ^162^ ^]^

Recent research has provided evidence for the involvement of neuroimmune units, integrating signals from the central nervous system, in the regulation of metabolism and obesity via the action of ILC2s. Acute cold challenge induces the expression of IL‐33 in PDGFRβ^+^ adipocyte precursor cells through β‐adrenergic receptors, leading to increased ILC2 accumulation and beige adipocyte accrual.^[^ ^163^ ^]^ ILC2s serve as the primary producers of IL‐5 and IL‐13 in adipose tissue, contributing to the expansion of eosinophils and macrophages.^[^ ^164^ ^]^ ILC2s and stromal cells can also produce methionine‐enkephalin (Met‐Enk) peptides that act on adipocytes through opioid growth factor receptor, promoting the upregulation of UCP1 expression and the induction of beiging in white adipose tissue.^[^ ^165^ ^]^ Additionally, the administration of exogenous IL‐33 to mice fed a high‐fat diet has been shown to restore the population of ILC2s and sympathetic innervation.^[^ ^166^ ^]^ Conversely, chemical sympathectomy reduces the frequency of ILC2s and eosinophils in iWAT, indicating the involvement of sympathetic regulation in shaping the immune environment.^[^ ^166^ ^]^ Sympathetic nerve terminals in mouse visceral adipose tissue have been shown to exert their influence on local mesenchymal stem cells through β2‐adrenergic receptors, leading to the modulation of glial‐derived neurotrophic factor release, indirectly influencing the activity of adipose tissue ILC2s via the neurotrophic factor receptor RET.^[^ ^165b^ ^]^ Retrograde tracing studies showed that these adipose tissue circuits are under the control of sympathetic fibers originating from the aorticorenal ganglion. These sympathetic fibers establish connections with higher‐order brain regions, including the paraventricular nucleus of the hypothalamus, thereby contributing to the regulation of adipose tissue ILC2 activity.^[^ ^165b^ ^]^

Eosinophils play a crucial role in adipose tissues as they are the primary producers of interleukin‐4 (IL‐4), which helps sustain the presence of adipose macrophages.^[^ ^167^ ^]^ Eosinophils, along with IL‐33 responsive ILC2s, are influenced by the levels of IL‐33 under the regulation of sympathetic signals in adipose tissues^[^ ^129^, ^166^ ^]^ Despite some controversy surrounding the abundance of eosinophils in murine adipose tissues during the transition from a lean to an obese state,^[^ ^166^, ^168^ ^]^ accumulating evidence suggests that eosinophils have beneficial effects in adipose tissues. Additionally, eosinophils have been implicated in promoting the beiging of WAT and enhancing thermogenesis, with their action being restricted by the transcriptional repressor Krüppel‐like factor 3 (KLF3).^[^ ^169^ ^]^

In recent studies, additional cell types have emerged as significant contributors to the neuroimmune regulation of adipose tissue. For instance, brown adipocytes have been found to produce IL‐6 in response to sympathetic activating during acute psychological stress, promoting hepatic gluconeogenesis to support fight‐or‐flight responses.^[^ ^170^ ^]^ T lymphocytes have also been implicated in enhancing sympathetic innervation of BAT by inducing the expression of TGFβ in adipocytes,^[^ ^171^ ^]^ highlighting the intricate crosstalk between the immune, adipocytes, and nervous systems in metabolic regulation.^[^ ^129a^ ^]^ Interestingly, social stress, which is known to activate the SNS, has been found to increase neutrophil accumulation in epididymal white adipose tissue (eWAT) and accelerate the development of insulin resistance in mice fed a high‐fat diet.^[^ ^172^ ^]^ Inhibition of neutrophil elastase, an enzyme released by neutrophils, has been shown to mitigate the impairment in insulin sensitivity observed in stressed mice.

Together, these findings collectively establish crucial crosstalk between the SNS and immune systems in adipose tissues, highlighting their mutual influence on maintaining nerve integrity and tissue function, ultimately exerting a significant impact on metabolism. Any disruption in this delicate neuroimmune interaction has the potential to impair nerve integrity and compromise tissue functionality. These insights emphasize the importance of understanding and preserving the balance and cooperation between the nervous and immune systems for optimal metabolic health.

## 6 Conclusions and Future Challenges

In this review, we illuminate the substantial body of evidence that underscores the pivotal role of sympathetic innervation in orchestrating metabolic processes within individual organs, including the pancreas, liver, intestine, and adipose tissue. Dysfunction within the immune microenvironment intricately correlates with organ pathologies characterized by sympathetic dysregulation. Gaining insight into the distinct patterns of sympathetic innervation that govern the temporal and spatial orchestration of immune and metabolic responses is of significant importance. It is worth noting that sympathetic‐regulated immune responses are not limited to metabolic organs but also extend to other conditions like chronic pain, cardiovascular disorders, and cancer. While this review primarily focuses on the metabolic organs, exploring the impact of sympathetic regulation on other organs, such as the cardiovascular system and tumor immunology, would be equally captivating.

As our comprehension of the SNS advances, so does the potential for innovative therapeutic strategies. Throughout the years, researchers and clinicians have recognized the therapeutic promise of targeting the SNS to manage various medical conditions. Techniques like sympathectomy, which involves surgical or chemical ablation of sympathetic nerve fibers or ganglia, and modulation of sympathetic activity through pharmacological agents or implanted electronic devices, have been employed to treat vascular disorders. Clinicians can selectively influence sympathetic outflow to specific organs or regions, offering potential therapeutic gains for conditions like hypertension, heart failure, and chronic pain syndromes. As we delve deeper into the intricate networks and mechanisms governing sympathetic regulation, manipulating post‐ganglionic sympathetic neurons to achieve control over metabolic and inflammatory processes will become a critical aspect of the therapeutic landscape in the foreseeable future.

Though substantial strides have been taken, several challenges persist in comprehending the intricacies of sympathetic neuroscience across multicellular networks and circuits. Ensuring cell type‐specific and organ‐specific investigations into sympathetic regulation and immune responses is imperative to accurately unveil the distinct roles of sympathetic nerves. This, in turn, allows us to effectively translate current insights into practical therapeutic strategies. Longitudinal studies and integration of multi‐omics data in both human subjects and animal models are poised to unveil the temporal dynamics of sympathetic‐ and immune‐mediated changes within metabolic organs and their relevance to disease progression. This burgeoning field of research holds the promise of yielding fruitful pathways for developing sympathoprotective therapeutic interventions, effectively combatting metabolic and inflammatory disorders.

## Conflict of Interest

The authors declare no conflict of interest.

## References

[1] a) C. Godinho‐Silva , F. Cardoso , H. Veiga‐Fernandes , Annu. Rev. Immunol. 2019, 37, 19; 30379595 10.1146/annurev-immunol-042718-041812

[2] Y. Zhao , B. Veysman , Biomedicines 2023, 11, 594. 36831130 10.3390/biomedicines11020594 PMC9952924

[3] a) R. F. Hampton , M. Jimenez‐Gonzalez , S. A. Stanley , Diabetologia 2022, 65, 1069; 35348820 10.1007/s00125-022-05691-9 PMC9205575

[4] a) M. Jimenez‐Gonzalez , R. Li , L. E. Pomeranz , A. Alvarsson , R. Marongiu , R. F. Hampton , M. G. Kaplitt , R. C. Vasavada , G. J. Schwartz , S. A. Stanley , Nat. Biomed. Eng. 2022, 6, 1298; 35835995 10.1038/s41551-022-00909-y PMC9669304

[5] J. Gromada , I. Franklin , C. B. Wollheim , Endocr. Rev. 2007, 28, 84. 17261637 10.1210/er.2006-0007

[6] R. Rodriguez‐Diaz , A. Caicedo , Best Pract. Res., Clin. Endocrinol. Metab. 2014, 28, 745. 25256769 10.1016/j.beem.2014.05.002

[7] a) H.‐J. Chien , T.‐C. Chiang , S.‐J. Peng , M.‐H. Chung , Y.‐H. Chou , C.‐Y. Lee , Y.‐M. Jeng , Y.‐W. Tien , S.‐C. Tang , Am. J. Physiol. 2019, 317, G694; 10.1152/ajpgi.00116.2019 31509431

[8] G. J. Taborsky , T. O. Mundinger , Endocrinology 2012, 153, 1055. 22315452 10.1210/en.2011-2040 PMC3384078

[9] P. Borden , J. Houtz , S. D. Leach , R. Kuruvilla , Cell Rep. 2013, 4, 287. 23850289 10.1016/j.celrep.2013.06.019 PMC3740126

[10] a) D. Hamamdzic , E. Duzic , J. D. Sherlock , S. M. Lanier , Am. J. Physiol. 1995, 269, E162; 7631772 10.1152/ajpendo.1995.269.1.E162

[11] a) B. Thorens , Diabetes, Obes. Metab. 2014, 16, 87; 25200301 10.1111/dom.12346

[12] T. H. Lindsay , K. G. Halvorson , C. M. Peters , J. R. Ghilardi , M. A. Kuskowski , G. Y. Wong , P. W. Mantyh , Neuroscience 2006, 137, 1417. 16388907 10.1016/j.neuroscience.2005.10.055

[13] K. E. Fasanella , J. A. Christianson , R. S. Chanthaphavong , B. M. Davis , J. Comp. Neurol. 2008, 509, 42. 18418900 10.1002/cne.21736 PMC2677067

[14] N. Martinez‐Sanchez , O. Sweeney , D. Sidarta‐Oliveira , A. Caron , S. A. Stanley , A. I. Domingos , Neuron 2022, 110, 3597. 36327900 10.1016/j.neuron.2022.10.017 PMC9986959

[15] C. J. Padro , V. M. Sanders , Semin. Immunol. 2014, 26, 357. 24486056 10.1016/j.smim.2014.01.003 PMC4116469

[16] a) H. Veiga‐Fernandes , D. Artis , Science 2018, 359, 1465; 29599230 10.1126/science.aap9598

[17] a) K. Amirshahrokhi , M. Ghazi‐Khansari , Cytokine 2012, 60, 522; 22901832 10.1016/j.cyto.2012.07.029

[18] Q. Mei , T. O. Mundinger , A. Lernmark , G. J. Taborsky Jr. , Diabetes 2002, 51, 2997. 12351439 10.2337/diabetes.51.10.2997

[19] T. O. Mundinger , Q. Mei , A. K. Foulis , C. L. Fligner , R. L. Hull , G. J. Taborsky Jr. , Diabetes 2016, 65, 2322. 27207540 10.2337/db16-0284 PMC4955989

[20] G. J. Taborsky , Q. Mei , D. J. Hackney , D. P. Figlewicz , R. Leboeuf , T. O. Mundinger , Diabetologia 2009, 52, 2602. 19798480 10.1007/s00125-009-1494-5

[21] M. Campbell‐Thompson , E. A. Butterworth , J. L. Boatwright , M. A. Nair , L. H. Nasif , K. Nasif , A. Y. Revell , A. Riva , C. E. Mathews , I. C. Gerling , D. A. Schatz , M. A. Atkinson , Sci. Rep. 2021, 11, 6562. 33753784 10.1038/s41598-021-85659-8 PMC7985489

[22] G. Christoffersson , S. S. Ratliff , M. G. Von Herrath , Sci. Adv. 2020, 6, eabb2878. 33052874 10.1126/sciadv.abb2878 PMC7531904

[23] G. J. Taborsky , Q. Mei , K. E. Bornfeldt , D. J. Hackney , T. O. Mundinger , Diabetes 2014, 63, 2369. 24608438 10.2337/db13-0778 PMC4066345

[24] W. Ying , Y. S. Lee , Y. Dong , J. S. Seidman , M. Yang , R. Isaac , J. B. Seo , B.‐H. Yang , J. Wollam , M. Riopel , J. Mcnelis , C. K. Glass , J. M. Olefsky , W. Fu , Cell Metab. 2019, 29, 457. 30595478 10.1016/j.cmet.2018.12.003 PMC6701710

[25] B. Calderon , J. A. Carrero , S. T. Ferris , D. K. Sojka , L. Moore , S. Epelman , K. M. Murphy , W. M. Yokoyama , G. J. Randolph , E. R. Unanue , J. Exp. Med. 2015, 212, 1497. 26347472 10.1084/jem.20150496 PMC4577842

[26] a) S. T. Ferris , P. N. Zakharov , X. Wan , B. Calderon , M. N. Artyomov , E. R. Unanue , J. A. Carrero , J. Exp. Med. 2017, 214, 2369; 28630088 10.1084/jem.20170074 PMC5551574

[27] a) H.‐S. Jun , C.‐S. Yoon , L. Zbytnuik , N. Van Rooijen , J.‐W. Yoon , J. Exp. Med. 1999, 189, 347; 9892617 10.1084/jem.189.2.347 PMC2192977

[28] K. Amirshahrokhi , A. Zohouri , Cytokine 2021, 138, 155394. 33310423 10.1016/j.cyto.2020.155394

[29] J. Sun , S. Qi , W. Liu , S. Xin , Y. Chang , Y. Yang , L. Zhou , Y. Zhang , Z. Chu , Pancreas 2015, 44, 1083. 26348466 10.1097/MPA.0000000000000410

[30] M. Guyot , T. Simon , F. Ceppo , C. Panzolini , A. Guyon , J. Lavergne , E. Murris , D. Daoudlarian , R. Brusini , H. Zarif , S. Abélanet , S. Hugues‐Ascery , J.‐L. Divoux , S. J. Lewis , A. Sridhar , N. Glaichenhaus , P. Blancou , Nat. Biotechnol. 2019, 37, 1446. 31712773 10.1038/s41587-019-0295-8

[31] a) H. V. Lin , D. Accili , Cell Metab. 2011, 14, 9; 21723500 10.1016/j.cmet.2011.06.003 PMC3131084

[32] a) C. Bernard , Med. J. Aust. 1965, 1, 119; 14248653

[33] T. Shimazu , A. Fukuda , Science 1965, 150, 1607. 4286322 10.1126/science.150.3703.1607

[34] C.‐X. Yi , S. E. La Fleur , E. Fliers , A. Kalsbeek , Biochim. Biophys. Acta 2010, 1802, 416. 20060897 10.1016/j.bbadis.2010.01.006

[35] a) H.‐R. Berthoud , Anat. Rec., Part A 2004, 280, 827; 10.1002/ar.a.20088 15382018

[36] J.‐M. Delalande , P. J. Milla , A. J. Burns , Anat. Rec., Part A 2004, 280, 848. 10.1002/ar.a.20090 15382016

[37] a) Y. Fukuda , M. Imoto , Y. Koyama , Y. Miyazawa , T. Hayakawa , J. Int. Med. Res. 1996, 24, 466; 8959530 10.1177/030006059602400603

[38] T. Shimazu , Nutrition 1996, 12, 65. 8838845 10.1016/0899-9007(96)00060-3

[39] R. Burcelin , M. Uldry , M. Foretz , C. Perrin , A. Dacosta , M. Nenniger‐Tosato , J. Seydoux , S. Cotecchia , B. Thorens , J. Biol. Chem. 2004, 279, 1108. 14581480 10.1074/jbc.M307788200

[40] T. O. Mundinger , G. J. Taborsky Jr. , Am. J. Physiol. Endocrinol. Metab. 2000, 278, E390. 10710492 10.1152/ajpendo.2000.278.3.E390

[41] A. Athari , K. Hanecke , K. Jungermann , Hepatology 1994, 20, 142. 8020883 10.1016/0270-9139(94)90146-5

[42] K. Beckh , H. Hartmann , K. Jungermann , FEBS Lett. 1982, 146, 69. 6754444 10.1016/0014-5793(82)80707-2

[43] F. R. Carreño , M. C. L. Seelaender , Cell Biochem. Funct. 2004, 22, 9. 14695648 10.1002/cbf.1047

[44] a) A. J. Krentz , D. Freedman , R. Greene , M. Mckinley , P. J. Boyle , D. S. Schade , Metabolism 1996, 45, 1214; 8843175 10.1016/s0026-0495(96)90238-3

[45] a) M. Kj˦r , J. Jurlander , S. Keiding , H. Galbo , P. Kirkegaard , E. Hage , J. Hepatol. 1994, 20, 97; 8201229 10.1016/s0168-8278(05)80473-8

[46] a) G. Perseghin , E. Regalia , A. Battezzati , S. Vergani , A. Pulvirenti , I. Terruzzi , D. Baratti , F. Bozzetti , V. Mazzaferro , L. Luzi , J. Clin. Invest. 1997, 100, 931; 9259593 10.1172/JCI119609 PMC508266

[47] a) M. Laryea , K. D. Watt , M. Molinari , M. J. Walsh , V. C. Mcalister , P. J. Marotta , B. Nashan , K. M. Peltekian , Liver Transplant. 2007, 13, 1109; 10.1002/lt.21126 17663411

[48] a) C. Estes , H. Razavi , R. Loomba , Z. Younossi , A. J. Sanyal , Hepatology 2018, 67, 123; 28802062 10.1002/hep.29466 PMC5767767

[49] Z. M. Younossi , A. B. Koenig , D. Abdelatif , Y. Fazel , L. Henry , M. Wymer , Hepatology 2016, 64, 73. 26707365 10.1002/hep.28431

[50] a) E. Sabath , A. Báez‐Ruiz , R. M. Buijs , Obes. Rev. 2015, 16, 871; 26214605 10.1111/obr.12308

[51] C. Hurr , H. Simonyan , D. A. Morgan , K. Rahmouni , C. N. Young , J. Physiol. 2019, 597, 4565. 31278754 10.1113/JP277994 PMC6716997

[52] C. Adori , T. Daraio , R. Kuiper , S. Barde , L. Horvathova , T. Yoshitake , R. Ihnatko , I. Valladolid‐Acebes , P. Vercruysse , A. M. Wellendorf , R. Gramignoli , B. Bozoky , J. Kehr , E. Theodorsson , J. A. Cancelas , B. Mravec , C. Jorns , E. Ellis , J. Mulder , M. Uhlén , C. Bark , T. Hökfelt , Sci. Adv. 2021, 7, eabg5733. 34290096 10.1126/sciadv.abg5733 PMC8294768

[53] a) G. W. Lambert , N. E. Straznicky , E. A. Lambert , J. B. Dixon , M. P. Schlaich , Pharmacol. Ther. 2010, 126, 159; 20171982 10.1016/j.pharmthera.2010.02.002

[54] J. F. Dillon , J. N. Plevris , J. Nolan , D. J. Ewing , J. M. Neilson , I. A. Bouchier , P. C. Hayes , Am. J. Gastroenterol. 1994, 89, 1544. 8079935

[55] M. Menacho‐Marquez , R. Nogueiras , S. Fabbiano , V. Sauzeau , O. Al‐Massadi , C. Dieguez , X. R. Bustelo , Cell Metab. 2013, 18, 199. 23931752 10.1016/j.cmet.2013.07.001

[56] a) Y. Miyazawa , Y. Fukuda , M. Imoto , Y. Koyama , H. Nagura , Am. J. Gastroenterol. 1988, 83, 1108; 3048081

[57] C. McKee , J. Soeda , E. Asilmaz , B. Sigalla , M. Morgan , N. Sinelli , T. Roskams , J. A. Oben , Biochem. Biophys. Res. Commun. 2013, 437, 597. 23850676 10.1016/j.bbrc.2013.07.005 PMC5226920

[58] a) C. Andre , D. Couton , J. Gaston , L. Erraji , L. Renia , P. Varlet , P. Briand , J. G. Guillet , Am. J. Physiol. 1999, 276, G647; 10070041 10.1152/ajpgi.1999.276.3.G647

[59] M. W. Robinson , C. Harmon , C. O'Farrelly , Cell Mol. Immunol. 2016, 13, 267. 27063467 10.1038/cmi.2016.3 PMC4856809

[60] a) W. G. Forssmann , S. Ito , J. Cell Biol. 1977, 74, 299; 406265 10.1083/jcb.74.1.299 PMC2109862

[61] H. B. Huan , X. D. Wen , X. J. Chen , L. Wu , L. L. Wu , L. Zhang , D. P. Yang , X. Zhang , P. Bie , C. Qian , F. Xia , Brain, Behav., Immun. 2022, 104, 222. 35365387 10.1016/j.bbi.2022.03.015

[62] R. C. Fonseca , G. S. Bassi , C. C. Brito , L. B. Rosa , B. A. David , A. M. Araujo , N. Nobrega , A. B. Diniz , I. C. G. Jesus , L. S. Barcelos , M. A. P. Fontes , D. Bonaventura , A. Kanashiro , T. M. Cunha , S. Guatimosim , V. N. Cardoso , S. O. A. Fernandes , G. B. Menezes , G. de Lartigue , A. G. Oliveira , Brain, Behav., Immun. 2019, 81, 444. 31271871 10.1016/j.bbi.2019.06.041 PMC7826199

[63] V. Racanelli , B. Rehermann , Hepatology 2006, 43, S54. 16447271 10.1002/hep.21060

[64] Z. Li , J. A. Oben , S. Yang , H. Lin , E. A. Stafford , M. J. Soloski , S. A. Thomas , A. M. Diehl , Hepatology 2004, 40, 434. 15368448 10.1002/hep.20320

[65] M. Minagawa , H. Oya , S. Yamamoto , T. Shimizu , M. Bannai , H. Kawamura , K. Hatakeyama , T. Abo , Hepatology 2000, 31, 907. 10733547 10.1053/he.2000.5850

[66] C. H. Wong , C. N. Jenne , W. Y. Lee , C. Leger , P. Kubes , Science 2011, 334, 101. 21921158 10.1126/science.1210301

[67] T. Liu , J. Li , Q. Li , Y. Liang , J. Gao , Z. Meng , P. Li , M. Yao , J. Gu , H. Tu , Y. Gan , Hepatology 2023, 78, 136. 36631003 10.1097/HEP.0000000000000239

[68] a) W. L. Neuhuber , G. Tiegs , Anat. Rec., Part A 2004, 280, 884; 10.1002/ar.a.20093 15382013

[69] a) D. Artis , Nat. Rev. Immunol. 2008, 8, 411; 18469830 10.1038/nri2316

[70] H. Selye , C. Fortier , Res. Publ. ‐ Assoc. Res. Nerv. Ment. Dis. 1949, 29, 3. 14854281

[71] D. Jacobowitz , J. Pharmacol. Exp. Ther. 1965, 149, 358. 4954685

[72] a) G. Gabella , M. Costa , Exper. Suppl. 1968, 24, 706; 10.1007/BF02138328 5705242

[73] A. E. Lomax , K. A. Sharkey , J. B. Furness , Neurogastroenterol. Motil. 2010, 22, 7. 19686308 10.1111/j.1365-2982.2009.01381.x

[74] a) L. Capurso , C. A. Friedmann , A. G. Parks , Gut 1968, 9, 678; 5717969 10.1136/gut.9.6.678 PMC1552872

[75] J. B. Furness , M. Costa , Ergeb. Physiol. 1974, 69, 2. 4365221

[76] N. O. Glebova , D. D. Ginty , J. Neurosci. 2004, 24, 743. 14736860 10.1523/JNEUROSCI.4523-03.2004 PMC6729267

[77] J. Hatch , Y. S. Mukouyama , Dev. Dyn. 2015, 244, 56. 25138596 10.1002/dvdy.24178 PMC8538805

[78] X. Niu , L. Liu , T. Wang , X. Chuan , Q. Yu , M. Du , Y. Gu , L. Wang , J. Neurosci. 2020, 40, 6691. 32690615 10.1523/JNEUROSCI.0309-20.2020 PMC7455214

[79] I. Brunet , E. Gordon , J. Han , B. Cristofaro , D. Broqueres‐You , C. Liu , K. Bouvree , J. Zhang , R. del Toro , T. Mathivet , B. Larrivee , J. Jagu , L. Pibouin‐Fragner , L. Pardanaud , M. J. Machado , T. E. Kennedy , Z. Zhuang , M. Simons , B. I. Levy , M. Tessier‐Lavigne , A. Grenz , H. Eltzschig , A. Eichmann , J. Clin. Invest. 2014, 124, 3230. 24937433 10.1172/JCI75181 PMC4071369

[80] a) M. Del Tacca , G. Soldani , C. Bernardini , E. Martinotti , M. Impicciatore , Eur. J. Pharmacol. 1982, 81, 255; 7117375 10.1016/0014-2999(82)90443-5

[81] A. Malcolm , M. Camilleri , L. Kost , D. D. Burton , S. L. Fett , A. R. Zinsmeister , Aliment. Pharmacol. Ther. 2000, 14, 783. 10848663 10.1046/j.1365-2036.2000.00757.x

[82] a) S. Schaak , D. Cussac , C. Cayla , J. C. Devedjian , R. Guyot , H. Paris , C. Denis , Gut 2000, 47, 242; 10896916 10.1136/gut.47.2.242 PMC1728001

[83] H. Duan , X. Cai , Y. Luan , S. Yang , J. Yang , H. Dong , H. Zeng , L. Shao , Front. Physiol. 2021, 12, 700129. 34335306 10.3389/fphys.2021.700129 PMC8317205

[84] a) E. A. Davis , W. Zhou , M. J. Dailey , Physiol. Rep. 2018, 6, e13745; 29932493 10.14814/phy2.13745 PMC6014443

[85] L. Hansen , S. Lampert , H. Mineo , J. J. Holst , Am. J. Physiol. Endocrinol. Metab. 2004, 287, E939. 15475512 10.1152/ajpendo.00197.2004

[86] a) A. L. Haber , M. Biton , N. Rogel , R. H. Herbst , K. Shekhar , C. Smillie , G. Burgin , T. M. Delorey , M. R. Howitt , Y. Katz , I. Tirosh , S. Beyaz , D. Dionne , M. Zhang , R. Raychowdhury , W. S. Garrett , O. Rozenblatt‐Rosen , H. N. Shi , O. Yilmaz , R. J. Xavier , A. Regev , Nature 2017, 551, 333; 29144463 10.1038/nature24489 PMC6022292

[87] C. Cailotto , L. M. Costes , J. van der Vliet , S. H. van Bree , J. J. van Heerikhuize , R. M. Buijs , G. E. Boeckxstaens , Neurogastroenterol. Motil. 2012, 24, 191. 22118533 10.1111/j.1365-2982.2011.01824.x

[88] L. M. Costes , G. E. Boeckxstaens , W. J. de Jonge , C. Cailotto , Organogenesis 2013, 9, 216. 23867810 10.4161/org.25646 PMC3896593

[89] A. N. Ananthakrishnan , C. N. Bernstein , D. Iliopoulos , A. Macpherson , M. F. Neurath , R. A. R. Ali , S. R. Vavricka , C. Fiocchi , Nat. Rev. Gastroenterol. Hepatol. 2018, 15, 39. 29018271 10.1038/nrgastro.2017.136

[90] a) S. M. Brierley , D. R. Linden , Nat. Rev. Gastroenterol. Hepatol. 2014, 11, 611; 25001973 10.1038/nrgastro.2014.103

[91] a) B. Ohlsson , G. Sundkvist , S. Lindgren , BMC Gastroenterol. 2007, 7, 33; 17697346 10.1186/1471-230X-7-33 PMC1978494

[92] a) K. Jacobson , K. McHugh , S. M. Collins , Gastroenterology 1997, 112, 156; 8978354 10.1016/s0016-5085(97)70230-0

[93] R. H. Straub , E. Antoniou , M. Zeuner , V. Gross , J. Scholmerich , T. Andus , J. Neuroimmunol. 1997, 80, 149. 9413271 10.1016/s0165-5728(97)00150-1

[94] F. Lechin , B. van der Dijs , C. L. Insausti , F. Gomez , S. Villa , A. E. Lechin , L. Arocha , O. Oramas , J. Clin. Pharmacol. 1985, 25, 219. 2860133 10.1002/j.1552-4604.1985.tb02828.x

[95] C. Blandizzi , M. Fornai , R. Colucci , F. Baschiera , G. Barbara , R. De Giorgio , F. De Ponti , M. C. Breschi , M. Del Tacca , Br. J. Pharmacol. 2003, 139, 309. 12770936 10.1038/sj.bjp.0705249 PMC1573848

[96] E. A. Mayer , H. J. Ryu , R. R. Bhatt , Mol. Psychiatry 2023, 28, 1451. 36732586 10.1038/s41380-023-01972-w PMC10208985

[97] R. M. Lovell , A. C. Ford , Clin. Gastroenterol. Hepatol. 2012, 10, 712. 22426087 10.1016/j.cgh.2012.02.029

[98] Y. Nasser , W. Ho , K. A. Sharkey , J. Comp. Neurol. 2006, 495, 529. 16498685 10.1002/cne.20898

[99] M. Di Giovangiulio , S. Verheijden , G. Bosmans , N. Stakenborg , G. E. Boeckxstaens , G. Matteoli , Front. Immunol. 2015, 6, 590. 26635804 10.3389/fimmu.2015.00590 PMC4653294

[100] a) S. Elsenbruch , W. C. Orr , Am. J. Gastroenterol. 2001, 96, 460; 11232691 10.1111/j.1572-0241.2001.03526.x

[101] a) A. Aggarwal , T. F. Cutts , T. L. Abell , S. Cardoso , B. Familoni , J. Bremer , J. Karas , Gastroenterology 1994, 106, 945; 8143999 10.1016/0016-5085(94)90753-6

[102] a) A. E. Bharucha , M. Camilleri , A. R. Zinsmeister , R. B. Hanson , Am. J. Physiol. 1997, 273, G997; 9374695 10.1152/ajpgi.1997.273.5.G997

[103] A. Sikander , S. V. Rana , S. K. Sharma , S. K. Sinha , S. K. Arora , K. K. Prasad , K. Singh , Clin. Chim. Acta 2010, 411, 59. 19833115 10.1016/j.cca.2009.10.003

[104] a) H. J. Kim , M. Camilleri , P. J. Carlson , F. Cremonini , I. Ferber , D. Stephens , S. McKinzie , A. R. Zinsmeister , R. Urrutia , Gut 2004, 53, 829; 15138209 10.1136/gut.2003.030882 PMC1774073

[105] N. Zou , H. Lv , J. Li , N. Yang , H. Xue , J. Zhu , J. Qian , Transl. Res. 2008, 152, 283. 19059163 10.1016/j.trsl.2008.10.002

[106] J. J. Sebastian Domingo , Med. Clin. 2022, 158, 76. 10.1016/j.medcli.2021.04.029 34238582

[107] B. B. Yoo , S. K. Mazmanian , Immunity 2017, 46, 910. 28636959 10.1016/j.immuni.2017.05.011 PMC5551410

[108] B. Sitkauskiene , R. Sakalauskas , Curr. Drug Targets: Inflammation Allergy 2005, 4, 157. 15853736 10.2174/1568010053586309

[109] I. Gabanyi , P. A. Muller , L. Feighery , T. Y. Oliveira , F. A. Costa‐Pinto , D. Mucida , Cell 2016, 164, 378. 26777404 10.1016/j.cell.2015.12.023 PMC4733406

[110] F. Matheis , P. A. Muller , C. L. Graves , I. Gabanyi , Z. J. Kerner , D. Costa‐Borges , T. Ahrends , P. Rosenstiel , D. Mucida , Cell 2020, 180, 64. 31923400 10.1016/j.cell.2019.12.002 PMC7271821

[111] T. Ahrends , B. Aydin , F. Matheis , C. H. Classon , F. Marchildon , G. C. Furtado , S. A. Lira , D. Mucida , Cell 2021, 184, 5715. 34717799 10.1016/j.cell.2021.10.004 PMC8595755

[112] D. R. Brown , L. D. Price , FEMS Immunol. Med. Microbiol. 2008, 52, 29. 18031537 10.1111/j.1574-695X.2007.00348.x

[113] a) S. Moriyama , J. R. Brestoff , A. L. Flamar , J. B. Moeller , C. S. N. Klose , L. C. Rankin , N. A. Yudanin , L. A. Monticelli , G. G. Putzel , H. R. Rodewald , D. Artis , Science 2018, 359, 1056; 29496881 10.1126/science.aan4829

[114] Z. Yin , Y. Zhou , H. R. Turnquist , Q. Liu , Trends Immunol. 2022, 43, 901. 36253275 10.1016/j.it.2022.09.006

[115] P. Wang , N. Kljavin , T. T. T. Nguyen , E. E. Storm , B. Marsh , J. Jiang , W. Lin , H. Menon , R. Piskol , F. J. de Sauvage , Cell Stem Cell 2023, 30, 1166. 37597516 10.1016/j.stem.2023.07.013

[116] a) M. Hadjifrangiskou , M. Kostakioti , S. L. Chen , J. P. Henderson , S. E. Greene , S. J. Hultgren , Mol. Microbiol. 2011, 80, 1516; 21542868 10.1111/j.1365-2958.2011.07660.x PMC3643513

[117] C. G. Moreira , R. Russell , A. A. Mishra , S. Narayanan , J. M. Ritchie , M. K. Waldor , M. M. Curtis , S. E. Winter , D. Weinshenker , V. Sperandio , mBio 2016, 7, e00826. 27273829 10.1128/mBio.00826-16 PMC4959670

[118] a) K. S. Cook , H. Y. Min , D. Johnson , R. J. Chaplinsky , J. S. Flier , C. R. Hunt , B. M. Spiegelman , Science 1987, 237, 402; 3299705 10.1126/science.3299705

[119] P. Cohen , S. Kajimura , Nat. Rev. Mol. Cell Biol. 2021, 22, 393. 33758402 10.1038/s41580-021-00350-0 PMC8159882

[120] S. Corvera , Annu. Rev. Physiol. 2021, 83, 257. 33566675 10.1146/annurev-physiol-031620-095446 PMC8091658

[121] a) A. K. Ramirez , S. N. Dankel , B. Rastegarpanah , W. Cai , R. Xue , M. Crovella , Y. H. Tseng , C. R. Kahn , S. Kasif , Nat. Commun. 2020, 11, 2117; 32355218 10.1038/s41467-020-16019-9 PMC7192917

[122] M. Giralt , F. Villarroya , Endocrinology 2013, 154, 2992. 23782940 10.1210/en.2013-1403

[123] a) T. J. Bartness , C. H. Vaughan , C. K. Song , Int. J. Obes. 2010, 34, S36; 10.1038/ijo.2010.182 PMC3999344 20935665

[124] J. Chi , Z. Lin , W. Barr , A. Crane , X. G. Zhu , P. Cohen , Elife 2021, 10, e64693; 33591269 10.7554/eLife.64693 PMC7990502

[125] C. Huesing , E. Qualls‐Creekmore , N. Lee , M. Francois , H. Torres , R. Zhang , D. H. Burk , S. Yu , C. D. Morrison , H. R. Berthoud , W. Neuhuber , H. Munzberg , J. Comp. Neurol. 2021, 529, 1465. 32935348 10.1002/cne.25031 PMC7960575

[126] A. Bagri , M. Tessier‐Lavigne , Adv. Exp. Med. Biol. 2002, 515, 13. 12613540

[127] A. Giordano , P. Cesari , L. Capparuccia , M. Castellucci , S. Cinti , J. Neurocytol. 2003, 32, 345. 14724377 10.1023/B:NEUR.0000011328.61376.bb

[128] S. Cinti , Compr. Physiol. 2018, 8, 1357. 30215863 10.1002/cphy.c170042

[129] a) E. E. Lin , E. Scott‐Solomon , R. Kuruvilla , Trends Neurosci. 2021, 44, 189; 33229051 10.1016/j.tins.2020.10.015 PMC7904596

[130] T. J. Bartness , Y. Liu , Y. B. Shrestha , V. Ryu , Front. Neuroendocrinol. 2014, 35, 473. 24736043 10.1016/j.yfrne.2014.04.001 PMC4175185

[131] R. B. S. Harris , Physiol. Behav. 2018, 190, 3. 28694155 10.1016/j.physbeh.2017.07.008 PMC5758439

[132] E. S. Bachman , H. Dhillon , C. Y. Zhang , S. Cinti , A. C. Bianco , B. K. Kobilka , B. B. Lowell , Science 2002, 297, 843. 12161655 10.1126/science.1073160

[133] a) M. Blaszkiewicz , J. W. Willows , C. P. Johnson , K. L. Townsend , Exp. Biol. Med. 2019, 8, 10; 10.3390/biology8010010 PMC6466238 30759876

[134] M. Francois , H. Torres , C. Huesing , R. Zhang , C. Saurage , N. Lee , E. Qualls‐Creekmore , S. Yu , C. D. Morrison , D. Burk , H. R. Berthoud , H. Munzberg , Ann. N. Y. Acad. Sci. 2019, 1454, 3. 31184376 10.1111/nyas.14119 PMC6810755

[135] a) M. Nechad , E. Ruka , J. Thibault , Comp. Biochem. Physiol., Part A: Physiol. 1994, 107, 381; 10.1016/0300-9629(94)90396-4 7907965

[136] P. Seale , S. Kajimura , W. Yang , S. Chin , L. M. Rohas , M. Uldry , G. Tavernier , D. Langin , B. M. Spiegelman , Cell Metab. 2007, 6, 38. 17618855 10.1016/j.cmet.2007.06.001 PMC2564846

[137] P. Cohen , J. D. Levy , Y. Zhang , A. Frontini , D. P. Kolodin , K. J. Svensson , J. C. Lo , X. Zeng , L. Ye , M. J. Khandekar , J. Wu , S. C. Gunawardana , A. S. Banks , J. P. Camporez , M. J. Jurczak , S. Kajimura , D. W. Piston , D. Mathis , S. Cinti , G. I. Shulman , P. Seale , B. M. Spiegelman , Cell 2014, 156, 304. 24439384 10.1016/j.cell.2013.12.021 PMC3922400

[138] R. P. Maickel , D. N. Stern , E. Takabatake , B. B. Brodie , J. Pharmacol. Exp. Ther. 1967, 157, 111. 6029477

[139] a) W. Wang , P. Seale , Nat. Rev. Mol. Cell Biol. 2016, 17, 691; 27552974 10.1038/nrm.2016.96 PMC5627770

[140] a) A. M. Cypess , L. S. Weiner , C. Roberts‐Toler , E. Franquet Elia , S. H. Kessler , P. A. Kahn , J. English , K. Chatman , S. A. Trauger , A. Doria , G. M. Kolodny , Cell Metab. 2015, 21, 33; 25565203 10.1016/j.cmet.2014.12.009 PMC4298351

[141] C. Tabuchi , H. S. Sul , Front. Endocrinol. 2021, 12, 595020. 10.3389/fendo.2021.595020 PMC8034539 33841324

[142] a) E. Winkler , M. Klingenberg , J. Biol. Chem. 1994, 269, 2508; 8300577

[143] C. E. Lyons , M. Razzoli , E. Larson , D. Svedberg , A. Frontini , S. Cinti , L. Vulchanova , M. Sanders , M. Thomas , A. Bartolomucci , FASEB J. 2020, 34, 2765. 31908033 10.1096/fj.201901361RR PMC7306786

[144] B. Cannon , J. Nedergaard , Physiol. Rev. 2004, 84, 277. 14715917 10.1152/physrev.00015.2003

[145] F. Villarroya , R. Cereijo , J. Villarroya , M. Giralt , Nat. Rev. Endocrinol. 2017, 13, 26. 27616452 10.1038/nrendo.2016.136

[146] T. Gnad , S. Scheibler , I. von Kugelgen , C. Scheele , A. Kilic , A. Glode , L. S. Hoffmann , L. Reverte‐Salisa , P. Horn , S. Mutlu , A. El‐Tayeb , M. Kranz , W. Deuther‐Conrad , P. Brust , M. E. Lidell , M. J. Betz , S. Enerback , J. Schrader , G. G. Yegutkin , C. E. Muller , A. Pfeifer , Nature 2014, 516, 395. 25317558 10.1038/nature13816

[147] a) M. Blaszkiewicz , J. W. Willows , A. L. Dubois , S. Waible , K. DiBello , L. L. Lyons , C. P. Johnson , E. Paradie , N. Banks , K. Motyl , M. Michael , B. Harrison , K. L. Townsend , PLoS One 2019, 14, e0221766; 31509546 10.1371/journal.pone.0221766 PMC6738614

[148] P. Wang , K. H. Loh , M. Wu , D. A. Morgan , M. Schneeberger , X. Yu , J. Chi , C. Kosse , D. Kim , K. Rahmouni , P. Cohen , J. Friedman , Nature 2020, 583, 839. 32699414 10.1038/s41586-020-2527-y

[149] A. Nakagomi , S. Okada , M. Yokoyama , Y. Yoshida , I. Shimizu , T. Miki , Y. Kobayashi , T. Minamino , npj Aging Mech. Dis. 2015, 1, 15009. 28721258 10.1038/npjamd.2015.9 PMC5514989

[150] A. Scanzano , M. Cosentino , Front. Pharmacol. 2015, 6, 171. 26321956 10.3389/fphar.2015.00171 PMC4534859

[151] a) S. P. Weisberg , D. McCann , M. Desai , M. Rosenbaum , R. L. Leibel , A. W. Ferrante Jr. , J. Clin. Invest. 2003, 112, 1796; 14679176 10.1172/JCI19246 PMC296995

[152] S. Yona , K. W. Kim , Y. Wolf , A. Mildner , D. Varol , M. Breker , D. Strauss‐Ayali , S. Viukov , M. Guilliams , A. Misharin , D. A. Hume , H. Perlman , B. Malissen , E. Zelzer , S. Jung , Immunity 2013, 38, 79. 23273845 10.1016/j.immuni.2012.12.001 PMC3908543

[153] M. S. Rahman , H. Jun , Front. Immunol. 2022, 13, 884126. 35493493 10.3389/fimmu.2022.884126 PMC9039244

[154] L. Tang , S. Okamoto , T. Shiuchi , C. Toda , K. Takagi , T. Sato , K. Saito , S. Yokota , Y. Minokoshi , Endocrinology 2015, 156, 3680. 26132918 10.1210/EN.2015-1096

[155] R. M. Pirzgalska , E. Seixas , J. S. Seidman , V. M. Link , N. M. Sanchez , I. Mahu , R. Mendes , V. Gres , N. Kubasova , I. Morris , B. A. Arus , C. M. Larabee , M. Vasques , F. Tortosa , A. L. Sousa , S. Anandan , E. Tranfield , M. K. Hahn , M. Iannacone , N. J. Spann , C. K. Glass , A. I. Domingos , Nat. Med. 2017, 23, 1309. 29035364 10.1038/nm.4422 PMC7104364

[156] C. J. O. O'Brien , E. R. Haberman , A. I. Domingos , Annu. Rev. Cell Dev. Biol. 2021, 37, 549. 34613819 10.1146/annurev-cellbio-120319-114106 PMC7614880

[157] C. D. Camell , J. Sander , O. Spadaro , A. Lee , K. Y. Nguyen , A. Wing , E. L. Goldberg , Y. H. Youm , C. W. Brown , J. Elsworth , M. S. Rodeheffer , J. L. Schultze , V. D. Dixit , Nature 2017, 550, 119. 28953873 10.1038/nature24022 PMC5718149

[158] A. Bertola , T. Ciucci , D. Rousseau , V. Bourlier , C. Duffaut , S. Bonnafous , C. Blin‐Wakkach , R. Anty , A. Iannelli , J. Gugenheim , A. Tran , A. Bouloumie , P. Gual , A. Wakkach , Diabetes 2012, 61, 2238. 22596049 10.2337/db11-1274 PMC3425417

[159] V. Elgazar‐Carmon , A. Rudich , N. Hadad , R. Levy , J. Lipid Res. 2008, 49, 1894. 18503031 10.1194/jlr.M800132-JLR200

[160] J. Liu , A. Divoux , J. Sun , J. Zhang , K. Clement , J. N. Glickman , G. K. Sukhova , P. J. Wolters , J. Du , C. Z. Gorgun , A. Doria , P. Libby , R. S. Blumberg , B. B. Kahn , G. S. Hotamisligil , G. P. Shi , Nat. Med. 2009, 15, 940. 19633655 10.1038/nm.1994 PMC2736875

[161] B. C. Lee , M. S. Kim , M. Pae , Y. Yamamoto , D. Eberle , T. Shimada , N. Kamei , H. S. Park , S. Sasorith , J. R. Woo , J. You , W. Mosher , H. J. Brady , S. E. Shoelson , J. Lee , Cell Metab. 2016, 23, 685. 27050305 10.1016/j.cmet.2016.03.002 PMC4833527

[162] T. Sasaki , K. Moro , T. Kubota , N. Kubota , T. Kato , H. Ohno , S. Nakae , H. Saito , S. Koyasu , Cell Rep. 2019, 28, 202. 31269440 10.1016/j.celrep.2019.06.016

[163] B. Shan , M. Shao , Q. Zhang , Y. A. An , L. Vishvanath , R. K. Gupta , Genes Dev. 2021, 35, 1333. 34531316 10.1101/gad.348762.121 PMC8494206

[164] a) A. B. Molofsky , J. C. Nussbaum , H. E. Liang , S. J. Van Dyken , L. E. Cheng , A. Mohapatra , A. Chawla , R. M. Locksley , J. Exp. Med. 2013, 210, 535; 23420878 10.1084/jem.20121964 PMC3600903

[165] a) J. R. Brestoff , B. S. Kim , S. A. Saenz , R. R. Stine , L. A. Monticelli , G. F. Sonnenberg , J. J. Thome , D. L. Farber , K. Lutfy , P. Seale , D. Artis , Nature 2015, 519, 242; 25533952 10.1038/nature14115 PMC4447235

[166] X. Ding , Y. Luo , X. Zhang , H. Zheng , X. Yang , X. Yang , M. Liu , J. Endocrinol. 2016, 231, 35. 27562191 10.1530/JOE-16-0229 PMC5003423

[167] D. Wu , A. B. Molofsky , H. E. Liang , R. R. Ricardo‐Gonzalez , H. A. Jouihan , J. K. Bando , A. Chawla , R. M. Locksley , Science 2011, 332, 243. 21436399 10.1126/science.1201475 PMC3144160

[168] a) W. R. Bolus , A. J. Kennedy , A. H. Hasty , Physiol. Rep. 2018, 6, e13919; 30488596 10.14814/phy2.13919 PMC6250927

[169] A. J. Knights , E. J. Vohralik , P. J. Houweling , E. S. Stout , L. J. Norton , S. J. Alexopoulos , J. J. Yik , H. Mat Jusoh , E. M. Olzomer , K. S. Bell‐Anderson , K. N. North , K. L. Hoehn , M. Crossley , K. G. R. Quinlan , Nat. Commun. 2020, 11, 2922. 32523103 10.1038/s41467-020-16758-9 PMC7286919

[170] H. Qing , R. Desrouleaux , K. Israni‐Winger , Y. S. Mineur , N. Fogelman , C. Zhang , S. Rashed , N. W. Palm , R. Sinha , M. R. Picciotto , R. J. Perry , A. Wang , Cell 2020, 182, 372. 32610084 10.1016/j.cell.2020.05.054 PMC7384974

[171] B. Hu , C. Jin , X. Zeng , J. M. Resch , M. P. Jedrychowski , Z. Yang , B. N. Desai , A. S. Banks , B. B. Lowell , D. Mathis , B. M. Spiegelman , Nature 2020, 578, 610. 32076265 10.1038/s41586-020-2028-z PMC7055484

[172] S. Motoyama , H. Yamada , K. Yamamoto , N. Wakana , K. Terada , M. Kikai , N. Wada , M. Saburi , T. Sugimoto , H. Kubota , D. Miyawaki , D. Kami , T. Ogata , M. Ibi , C. Yabe‐Nishimura , S. Matoba , Cells 2020, 9, 996. 32316265 10.3390/cells9040996 PMC7226953

